# Tribology of GA and GI Steel Sheets in Forming: Coating-Failure Mechanisms, Evaluation Methods, and Governing Factors

**DOI:** 10.3390/ma19173683

**Published:** 2026-08-30

**Authors:** Tianqi Liu, Mah-E-Rukh Mustafa, Jia Cui, Ruidi Chen, Xing Wei, Biao Ma, Xianglin Zhang

**Affiliations:** 1 State Key Laboratory of Materials Processing and Die & Mould Technology, School of Materials Science and Engineering, Huazhong University of Science and Technology, Wuhan 430074, Chinamahee.rukh@gmail.com (M.-E.-R.M.);; 2 R&D Central Research Institute, Baoshan Iron & Steel Co., Ltd., 28# Yejin Road, Qingshan, Wuhan 430080, China

**Keywords:** galvanized (GI) sheet, galvannealed (GA) sheet, failure mechanism, sheet metal forming, friction and wear

## Abstract

This review provides a critical overview of wear and coating failure in galvanized (GI) and galvannealed (GA) steel-sheet forming. Galvanized (GI) and galvannealed (GA) steel sheets are extensively used in automotive sheet-metal forming because they combine corrosion protection with acceptable formability. However, their zinc-based coatings are highly susceptible to friction- and deformation-induced damage during forming operations, which can lead to coating degradation, surface deterioration, galling, unstable production, and accelerated tool wear. Owing to substantial differences in coating microstructure and mechanical response, GI and GA sheets exhibit distinct tribological behaviors and failure modes under comparable forming conditions. GI coatings, characterized by a softer Zn-rich outer layer, are more prone to smearing, adhesion, and galling, whereas GA coatings, composed of brittle Fe–Zn intermetallic phases, are more susceptible to cracking, flaking, powdering, and debris-induced abrasion. This review provides a critical overview of wear and coating failure in GI and GA steel-sheet forming. First, the intrinsic characteristics of GI and GA coatings are compared in terms of microstructure, surface morphology, and mechanical and tribological properties. Second, widely used laboratory methods for friction and wear evaluation are reviewed, including strip-drawing, twist compression, draw-bead, tensile strip, deep-drawing, and U-channel forming tests, with emphasis on the contact conditions and failure mechanisms they can reveal. Third, the dominant damage mechanisms are discussed, including adhesive wear, abrasive wear, coating fracture, material transfer, and galling. Finally, the effects of tool properties, lubrication, forming parameters, and finite-element simulation on coating failure are critically assessed. The available evidence indicates that coating failure in GI and GA sheets is governed by the coupled interaction among coating microstructure, contact pressure, tool-surface condition, lubrication state, and deformation path. However, current studies remain fragmented, and an integrated understanding of coating-damage evolution under industrially relevant forming conditions is still lacking. Future research should therefore focus on coating-specific tribological models, in situ and multiscale characterization, and coupled experimental–numerical strategies for process optimization while preserving coating integrity.

## 1. Introduction

Galvanized steel sheets are widely used in the automotive, construction, and general manufacturing sectors because their zinc coatings provide sacrificial protection, resulting in excellent corrosion resistance, good formability, durability, and cost-effectiveness [1]. With the rapid growth of the new-energy vehicle industry, the demand for high-performance coated steels has increased further, driven by the need for lightweight body structures and reliable battery-pack protection. As a result, galvanized steel products are expected to maintain strong market growth in the coming years.

Among galvanized steel products, hot-dip galvanized (GI) and hot-dip galvannealed (GA) steel sheets are the most important materials for sheet metal forming applications, particularly in the automotive industry [2,3,4]. GI coatings generally offer better corrosion resistance at lower cost, whereas GA coatings provide superior paintability and weldability. Owing to differences in coating structure, surface morphology, and mechanical properties, GI and GA sheets exhibit distinct tribological behaviors and failure modes during forming operations. Both materials are susceptible to coating-related failures, including cracking, flaking, delamination, galling, and powdering, which can impair corrosion resistance and surface quality, accelerate tool wear, reduce process stability, and increase production costs. These failures arise from the coupled effects of coating characteristics, tool properties, lubrication conditions, and forming parameters. Therefore, understanding the differences between GI and GA coatings and clarifying their failure mechanisms are essential for improving forming reliability and optimizing manufacturing processes [5].

Although a considerable number of studies have investigated the behavior of GI AND GA sheets from different perspectives, including coating properties, contact conditions, tool materials, and process parameters [6,7], the existing literature remains fragmented. Material characteristics, test methods, wear phenomena, and process variables are often discussed separately, making it difficult to establish a coherent understanding of how coating structure, tribological response, and forming conditions interact to govern failure behavior. In particular, comparative analyses that directly relate the distinct characteristics of GI and GA coatings to their tribological performance and coating-failure mechanisms remain limited. An integrated review is therefore needed, especially one that connects evaluation methods, wear mechanisms, and process variables within a unified framework.

**Figure 1:**
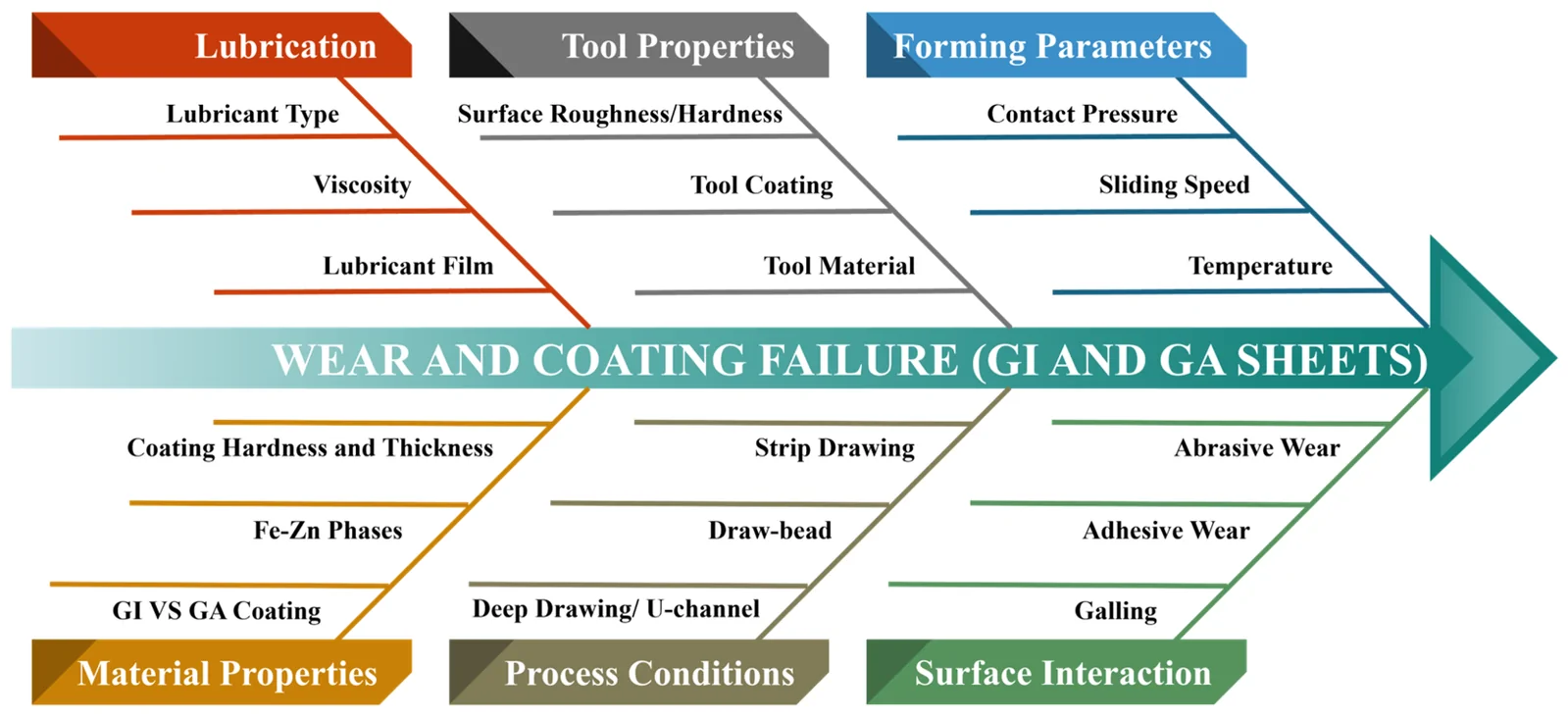
Schematic overview of the key aspects of tribology in GI and GA during sheet-metal forming.

To provide a comprehensive overview of the factors governing coating degradation, Figure 1 presents an Ishikawa (cause-and-effect) diagram summarizing the principal causes of wear and coating failure in GI and GA steel sheets during sheet metal forming. The diagram organizes the major contributing factors into six categories, including material properties, tool characteristics, lubrication conditions, forming parameters, surface interactions, and evaluation methods. It illustrates that coating failure is not the result of a single mechanism but rather arises from the complex interaction of multiple tribological and process-related factors. This framework serves as a conceptual guide for the subsequent sections of this review, in which each category is discussed in detail.

Accordingly, this review provides a concise and systematic overview of coating failure in GA and GI steel sheets during sheet-metal forming. First, the fundamental characteristics of GI and GA coatings are compared, including coating structure, surface morphology, and mechanical and tribological properties. Second, commonly used laboratory methods for friction and wear evaluation are reviewed, together with the failure mechanisms they are able to reveal. Third, the dominant wear and coating-damage mechanisms in GI and GA forming are discussed. Finally, the key factors governing coating failure—including tool properties, lubrication conditions, forming parameters, and finite element simulation—are critically assessed. On this basis, the major conclusions, current research gaps, and future directions for wear control and process optimization are outlined.

## 2. Characteristics of GI and GA Coatings Relevant to Forming Tribology

The distinct failure behaviors of GI and GA steel sheets during forming originate primarily from differences in coating structure, surface morphology, and mechanical and tribological characteristics. A comparative understanding of these intrinsic features is therefore essential for explaining why the two coating systems respond differently under similar contact and deformation conditions.

**Figure 2:**
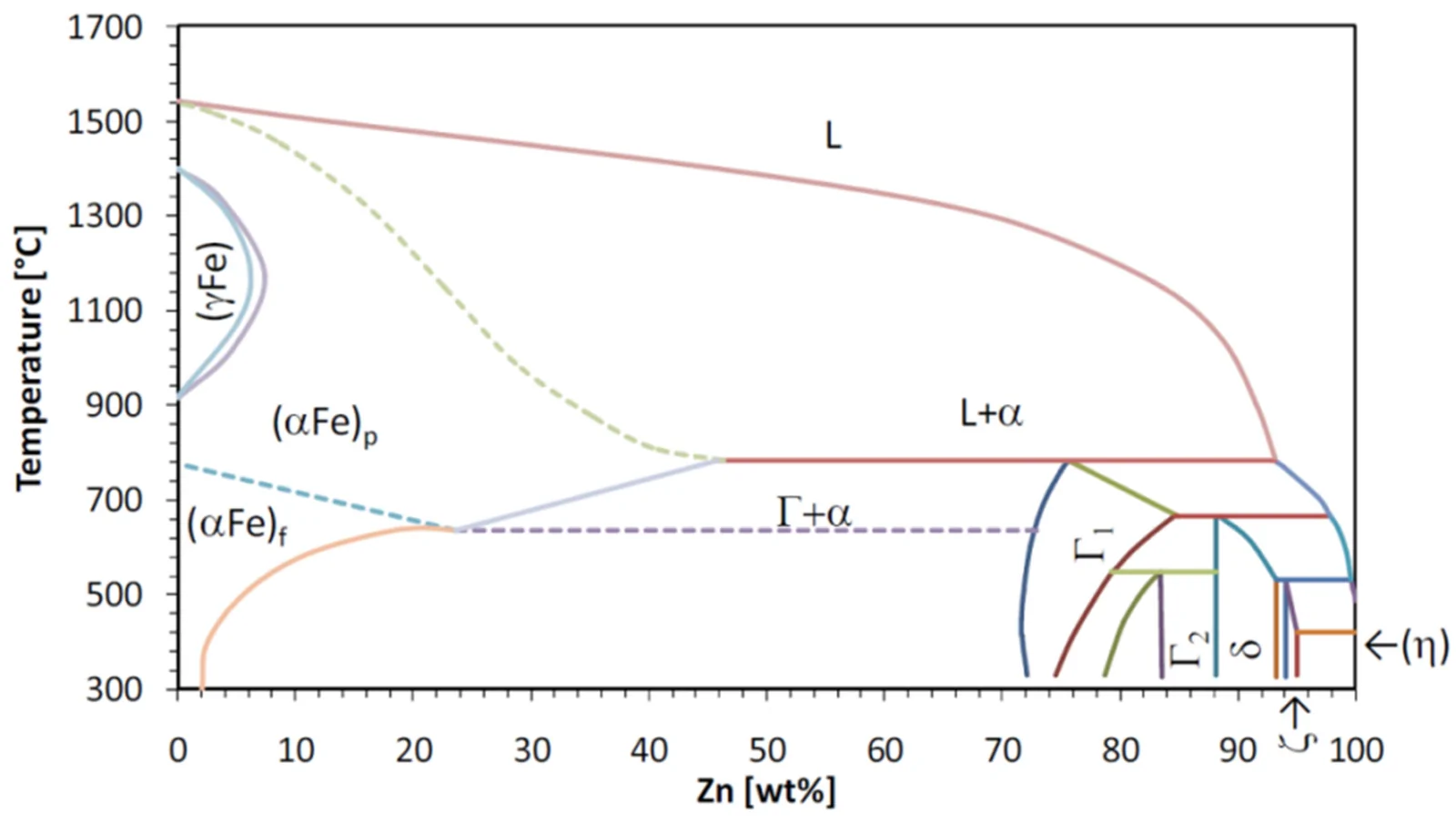
Fe–Zn binary equilibrium phase diagram [8].

Figure 2 shows the Fe Zn binary equilibrium phase diagram, with the enlarged area on the right clearly displaying the phases related to hot-dip galvanizing and key constant temperature reaction temperatures, including four types of iron zinc intermetallic compounds: Gamma 1 (Γ_1_), Gamma (Γ), Delta (δ), and Zeta (ζ). 782 °C, 665 °C, 550 °C, 530 °C, and 425 °C correspond to important phase transitions in the system in sequence. The GI coating is mainly composed of pure zinc phase; the GA coating undergoes alloying heat treatment, and Fe diffuses into the zinc layer, sequentially generating the brittle intermetallic phases mentioned above. The significant differences in hardness and plasticity of various phases determine the distinct stamping friction behavior between GI and GA coatings, which is the thermodynamic basis for explaining the mechanism of zinc layer peeling and bite failure during the forming process of the two galvanized steel plates.

**Figure 3:**
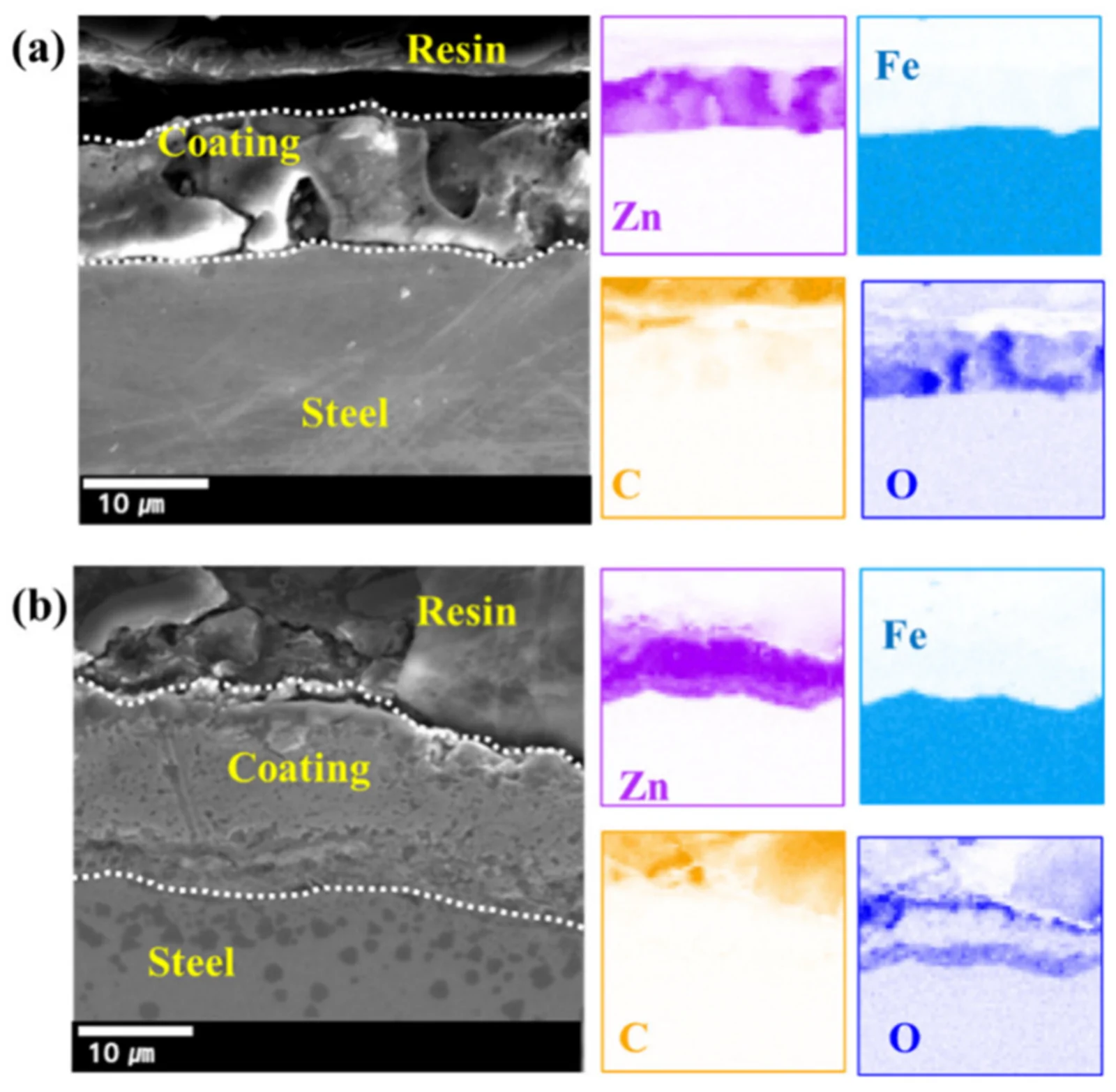
The cross-sectional view and EDS images of the four as-received specimen surfaces: (**a**) TS340-GI, (**b**) TS980-GI [10].

GI steel is produced by hot-dip galvanizing, in which the steel substrate is immersed in molten zinc to form a zinc-based protective coating that provides sacrificial corrosion protection [9]. Its microstructure typically consists of a steel substrate, a thin interfacial inhibition layer, and a zinc-rich outer coating. As shown in Figure 3, the GI coating is composed mainly of Zn, whereas Fe is concentrated in the substrate and a thin Al-rich layer is present at the interface. This structure gives GI steel good ductility and deformability, allowing the coating to accommodate contact-induced deformation during forming. However, the softness of the outer Zn-rich layer also makes GI coatings more susceptible to smearing, adhesion, and galling under severe frictional contact.

**Figure 4:**
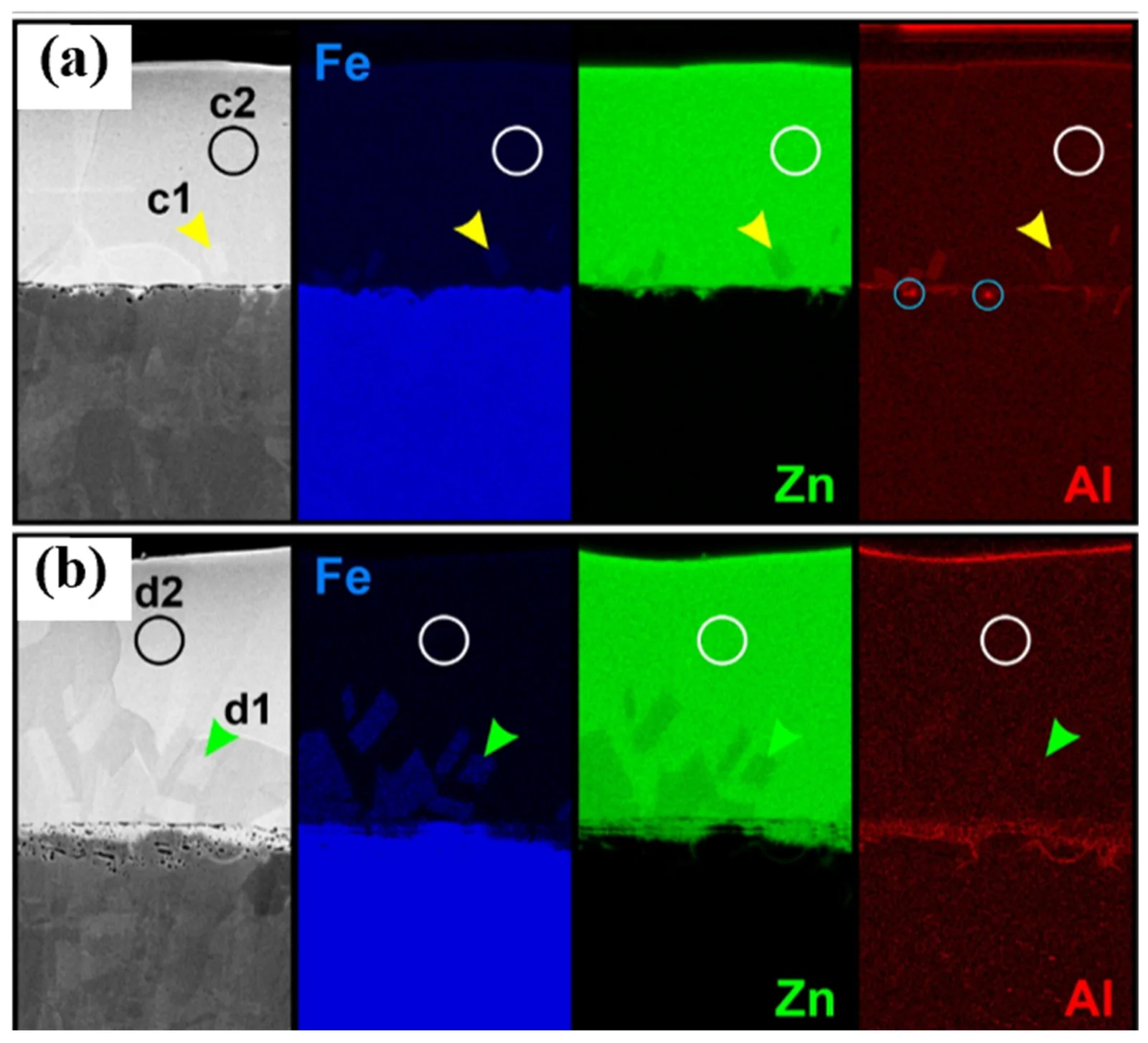
SEM images and corresponding EDX elemental mappings for (**a**) as-galvanized and (**b**) galvannealed (after 30 s) [13]. Yellow arrows denote Al-rich regions at the steel-coating interface; green arrows indicate characteristic interfacial reaction sites; blue circles mark discrete Al-enriched spots along the interface.

In contrast, GA steel is obtained by post-heat treatment of hot-dip galvanized coatings, during which the original Zn layer is transformed into a series of Zn–Fe intermetallic phases, mainly Γ, δ, and ζ [11]. Compared with GI, the GA coating therefore has a more complex alloyed structure, and the distribution, morphology, and fraction of these intermetallic phases strongly affect its forming performance. Surface observations further show that GA coatings are generally rougher and more porous than GI coatings, and that their roughness can increase with variations in processing parameters such as Al content in the zinc bath and alloying time [12]. EDS analysis of the GA cross-section indicates a more uniform distribution of Zn throughout the coating, together with detectable Fe within the coating and only a very limited amount of Al at the interface, as shown in Figure 4. These structural features make GA coatings harder and more resistant to superficial scratching during handling than GI coatings. These microstructural characteristics endow GA coatings with higher hardness and better resistance to surface scratching during handling compared to GI coatings, but they also render the coating more brittle and less capable of accommodating plastic deformation. As a result, GA steel is more prone to cracking, flaking, and powdering during forming, whereas GI steel more often exhibits deformation-dominated damage modes such as smearing and adhesion. In this sense, the distinction between GI and GA is not merely compositional; rather, it reflects a fundamental difference in how coating structure and surface morphology govern tribological response and ultimately determine failure mode.

The mechanical performance of GI and GA sheets during forming should be distinguished between the macroscopic load-bearing function of the steel substrate and the local integrity of the coating–substrate system. In most forming operations, the steel substrate carries the major tensile, bending, and global deformation load, whereas the zinc-based coating is subjected to localized normal pressure, tangential shear, asperity indentation, and strain incompatibility with the substrate. Accordingly, coating failure cannot be evaluated from coating hardness alone; it is governed jointly by coating ductility or brittleness, phase constitution, coating thickness, substrate support, interfacial adhesion, contact geometry, and lubrication conditions.

For GI sheets, the Zn-rich coating is relatively soft and can deform plastically under normal contact. A semi-analytical contact model developed for coated sheets showed that a softer zinc coating promotes asperity flattening and can result in a larger real contact area than that of uncoated steel under the same normal load [14]. This deformation accommodation can reduce local stress concentration during moderate loading; however, it may also facilitate local ploughing, smearing, and material transfer when the contact pressure is high or lubrication breaks down. U-channel forming studies have shown that GI sheets are particularly susceptible to surface deterioration associated with scratching and galling, and that the severity of this damage depends strongly on die hardness and surface condition [10,15,16]. In addition, crystal-plasticity simulations of hot-dip zinc coatings have demonstrated that substrate constraint produces heterogeneous stress states within the coating during tensile deformation, indicating that local coating response must be considered together with the mechanical support provided by the steel substrate [17].

For GA sheets, the Fe–Zn intermetallic coating provides higher hardness but lower deformation tolerance than the Zn-rich GI coating. The mechanical incompatibility among the intermetallic phases and the steel substrate promotes local stress concentration during deformation. Experimental and numerical studies have shown that cracks may initiate in brittle Fe–Zn phases during bending and tensile deformation, followed by crack propagation, fragmentation, powdering, or interfacial exfoliation under more severe loading conditions [12,18,19,20,21]. He et al. [20], for example, reported that the Γ phase in GA coatings exhibits high strength but very limited plastic accommodation, which contributes to early crack initiation during bending. Park et al. [21] further showed that combined tensile deformation and frictional shear in bead-slide forming can lead to localized interfacial exfoliation of GA coatings. Therefore, GA coatings may better resist mild surface indentation than GI coatings, but they are more sensitive to localized deformation and shear stresses that exceed the fracture resistance of the brittle intermetallic layers.

The currently reviewed literature provides substantial evidence for the effects of contact pressure, bending, tensile deformation, and frictional shear on GI and GA coating integrity. However, direct and systematic comparisons of GI and GA coatings during blanking, trimming, piercing, or other shearing operations remain limited. Consequently, the coating response near a cut edge should not be inferred solely from bulk sheet strength or coating hardness. Future studies should quantify coating damage after shearing using indicators such as coating-loss width, crack density near the cut edge, exposed-substrate area, burr morphology, and the amount and composition of detached debris. Such work is needed to establish whether the deformation-dominated damage of GI and the brittle fracture-dominated damage of GA persist under industrial cutting and trimming conditions.

Overall, the contrasting tribological behaviors of GI and GA sheets can be understood as a direct consequence of their coating architecture. GI coatings, with their softer Zn-rich outer layer and better deformability, tend to alleviate local stress concentration but are more vulnerable to adhesive transfer and galling. By contrast, GA coatings, which are dominated by harder and more brittle Zn–Fe intermetallic phases, are less susceptible to plastic material transfer but more prone to cracking, fragmentation, and debris generation. These intrinsic differences form the mechanistic basis for the distinct wear and coating-failure behaviors discussed in the following sections. To further clarify the mechanistic differences between the two coating systems, Table 1 summarizes their intrinsic characteristics and the corresponding tribological implications in sheet-metal forming.

## 3. Experimental Approaches for Evaluating Friction, Wear, and Coating Failure

Laboratory tribological testing provides an important link between the intrinsic properties of GI and GA coatings and their failure behavior during sheet-metal forming. Because sheet–tool interaction is governed by coupled effects of contact pressure, sliding, bending–unbending deformation, lubrication, and tool-surface condition, no single test can fully reproduce all aspects of practical forming. Accordingly, a variety of laboratory methods have been developed to simulate forming-related stress and friction conditions and to evaluate the effects of die materials, die coatings, lubricants, and tool hardness on friction, surface damage, and coating degradation. These methods can be broadly divided into friction-oriented tests, which are mainly used to measure the coefficient of friction and detect early interfacial damage, and process-simulation tests, which better reproduce practical-forming stress states and are therefore more suitable for assessing coating failure under realistic deformation conditions. Their significance lies not only in friction measurement, but also in revealing the dominant tribological and failure mechanisms that govern the formability of GI and GA steel sheets.

### 3.1. Laboratory Methods for Friction and Wear Evaluation

**Figure 5:**
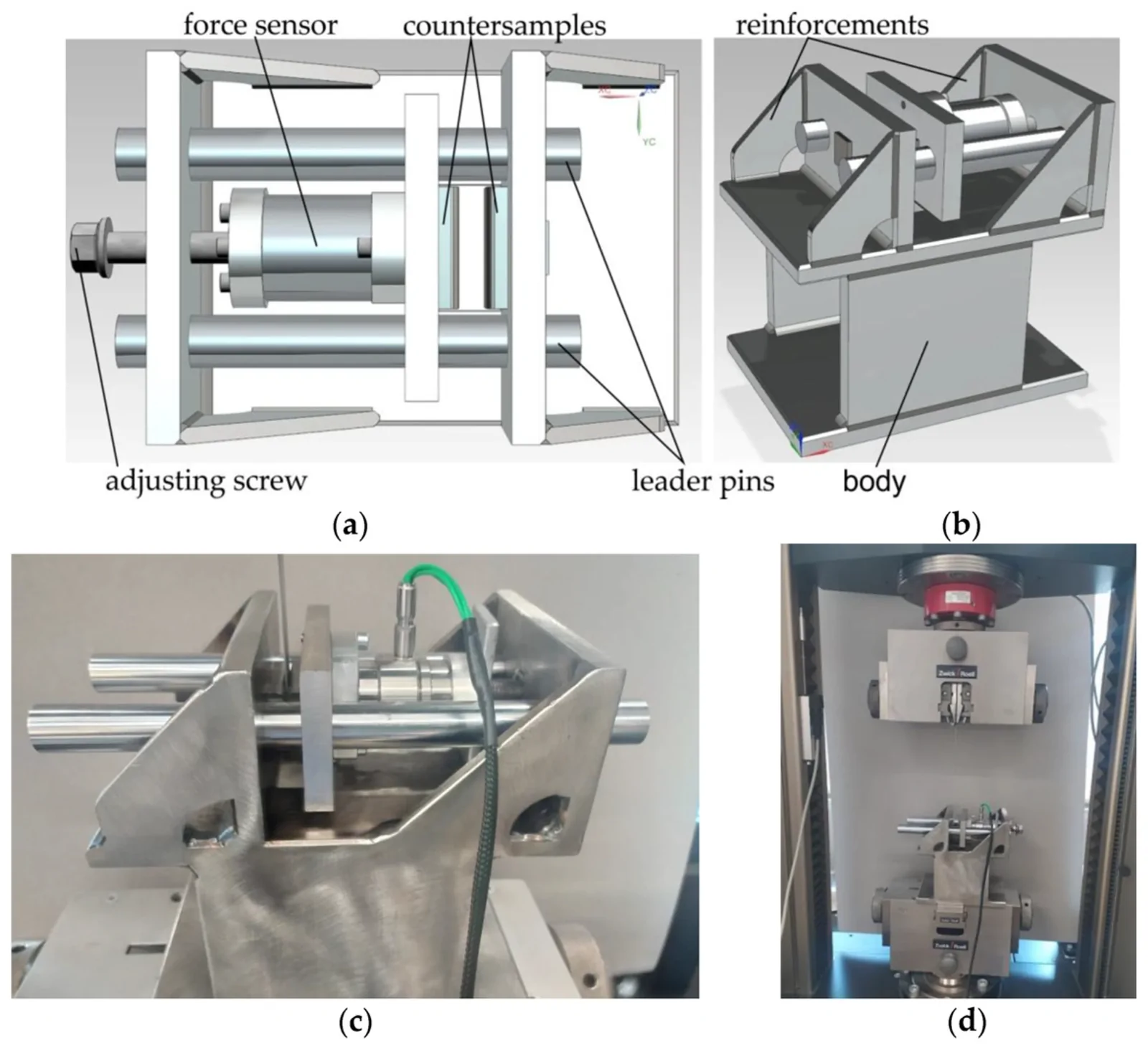
Geometric model of the friction tester in (**a**) top and (**b**) isometric views, (**c**) an actual photo and (**d**) friction tester mounted in Zwick/Roell Z100 testing machine (ZwickRoell GmbH & Co. KG, Ulm, Germany) [22].

Friction-oriented laboratory tests are primarily used to quantify interfacial friction and examine the early stages of lubricant breakdown, surface damage, and material transfer. Among them, the strip-drawing test is one of the most widely used methods in sheet metal forming tribology. As illustrated in Figure 5, Szewczyk et al. [22] employed this test to evaluate the frictional behavior of 0.8 mm-thick DC04 steel sheets. In the strip-drawing test, a metal strip is pulled between two counter-surfaces with flat working faces. Owing to its simple configuration, straightforward force measurement, and relatively high accuracy in determining the average coefficient of friction from the frictional forces acting on the two contact surfaces, this test is widely regarded as a reliable method for tribological assessment in sheet-metal forming. However, because two separate contact regions are involved and strip bending may occur during the test, some uncertainty may be introduced into the measured friction coefficient.

The twist compression test (TCT) is another laboratory method commonly used to evaluate the effects of lubricants and additives [23]. Kim et al. [24] applied the TCT to determine the time-dependent friction behavior of different lubricant systems in stamping processes involving various tool coatings and GI and GA sheet materials. This method is particularly useful because it enables simultaneous monitoring of friction and interface temperature, while also providing insight into lubricant performance, galling initiation, and surface-defect development under forming-relevant contact conditions. Another important friction-related test is the draw-bead test, which reproduces the bending–unbending and sliding behavior characteristic of sheet-metal forming and allows the coefficient of friction to be evaluated during sheet sliding against the die [25]. When appropriate equipment is available, the draw-bead test can be performed using conventional electromechanical or servo-hydraulic tensile testing machines, enabling direct measurement of forces associated with the forming process.

In addition, the tensile strip test is widely used to evaluate friction and to provide further insight into lubricant behavior and the sliding of the sheet over a punch surface [26]. In this method, the sheet strip is drawn over a cylindrical pin to simulate drawing and stretching conditions, and the coefficient of friction is calculated from the measured strip tension using the Capstan model [27]. Post-test examination of the pin surface can further reveal the extent of wear and material transfer. Collectively, these friction-oriented methods are particularly valuable for isolating the effects of lubrication, tool-surface condition, and contact geometry on the early tribological response of galvanized sheets.

Process-simulation tests, by contrast, are designed to reproduce more realistically the deformation paths and stress states encountered in industrial stamping. The strip-drawing test, originally developed by Hirasaka and Nishimura, has also been used in a simulative sense to reproduce forming-related loads and contact conditions using geometrically simple tools [28]. Kim et al. [29] applied this method, to investigate the behavior of different grades of advanced high-strength steels (AHSSs), including DP980 GI, DP600 GI, and DP590 GA, under varying die materials, die coatings, and lubrication conditions. Another commonly used process-simulation method is the deep-drawing cup test, in which a sheet blank is drawn into a cup-shaped part. Altan et al. [30] employed this test to evaluate lubricant performance by measuring draw-in length and failure limits under different blank-holder forces. This approach enables lubricant behavior to be assessed under controlled forming stresses. In addition, the U-channel forming test reproduces key features of practical stamping operations, including bending, deep drawing, and stretching. Hou et al. [31] used this method to examine the effect of stress state on galling behavior during stamping, while Yu et al. [15] applied it to investigate the surface damage of GA and GI steels under different tool hardness conditions.

Taken together, the available laboratory methods provide complementary approaches for evaluating the tribological behavior of GI and GA steel sheets under forming conditions. Friction-oriented tests, such as strip-drawing, TCT, draw-bead, and tensile-strip tests, are particularly useful for quantifying the coefficient of friction and identifying the early stages of lubricant breakdown, galling, and material transfer. By contrast, process-simulation methods, including deep drawing and U-channel forming, more closely reproduce the stress states encountered in practical stamping and are therefore better suited to assessing coating damage and wear under realistic deformation conditions. The choice of test method should therefore be guided by the specific objective of the study, whether it is friction characterization, lubricant evaluation, galling assessment, or coating-failure analysis.

To further compare the tribological responses of GI and GA sheets, Table 2 summarizes the limited quantitative results reported under identical or closely comparable test conditions. Although directly comparable GI/GA datasets remain limited, the available results show clear coating-dependent trends. Under comparable conditions, GA generally exhibits higher surface roughness and more fracture-related damage, whereas GI is more prone to adhesion and galling. For example, Kim et al. [10] reported Ra values of 0.85–0.92 μm for GI and 1.16–1.18 μm for GA, while the COF ranges partly overlapped (0.059–0.180 for GI and 0.125–0.177 for GA). Similar trends were observed in U-channel forming tests, where GA showed greater surface deterioration after repeated forming. However, matched data for wear rate and crack density remain scarce, highlighting the need for standardized GI/GA comparative studies.

### 3.2. Major Failure Mechanisms in Gi and Ga Sheet Forming

Results from these laboratory methods indicate that the failure behavior of GI and GA sheets during forming is governed primarily by the coupled evolution of adhesive wear, abrasive wear, and coating fracture. These mechanisms do not occur independently; rather, they often interact and intensify one another under practical forming conditions.

Adhesive wear refers to the transfer of material from one surface to another during relative motion, usually when the weaker material fails at the contact interface [33]. The transferred material may subsequently become strain-hardened and then act as an abrasive third body, thereby promoting further surface damage [33]. With repeated forming, the transfer layer can grow progressively, causing surface scratching and eventually evolving into severe adhesive wear with significant macroscopic damage [34]. According to wear severity, adhesive wear can be classified as scoring, galling, scuffing, and seizure. Scoring is characterized by severe scratches and grooves along the sliding direction, typically resulting from lubrication breakdown, metal-to-metal contact, and repeated tearing and welding of transferred material [35]. Galling is the most common adhesive wear mode in sheet metal forming and involves localized welding and tearing, leading to material transfer from the workpiece to the die surface, surface roughening, and protrusion formation [24,33]. It is strongly affected by tool and sheet properties, lubrication, contact conditions, and especially tool-surface roughness [36,37,38]. Scuffing is a sudden and catastrophic adhesive failure caused by localized solid-phase welding under high contact pressure and sliding speed [39,40,41]. Seizure is the most severe form, in which the contacting surfaces bind together as a result of extensive.

Abrasive wear occurs when a harder surface or loose hard particles remove material from a softer counter-surface through cutting or scratching [34]. Although it is not usually the dominant wear mode in sheet-metal forming, it can arise when the tool surface is excessively rough or when wear debris generated by adhesive wear acts as a third-body abrasive during sliding contact [33]. Abrasive wear can be classified into two-body abrasion, caused by direct asperity–surface interaction, and three-body abrasion, caused by loose particles trapped at the interface [34]. According to Kato [42], abrasive friction in steel mainly involves three mechanisms: cutting, wedging, and ploughing. Cutting removes material directly in the form of microchips, leading to severe material loss. Wedging forces material upward and outward from the groove, which may promote crack initiation and fatigue damage. Ploughing mainly causes plastic displacement of material to the sides of the groove, forming ridges and surface grooves; subsequent material detachment may occur under repeated contact [5,42]. In GI and GA sheet forming, abrasive wear is important because both the soft Zn coating in GI steel and the hard Zn–Fe intermetallic phases in GA steel can influence scratching behavior and interfacial wear in different ways. Consequently, abrasive wear can shorten tool life and deteriorate the surface quality of formed parts.

In addition to adhesive and abrasive wear, coating fracture is a key damage mode in galvanized sheet forming, especially for GA coatings. Owing to the brittle nature of Fe–Zn intermetallic phases, GA coatings are more prone to cracking, flaking, and powdering under combined contact pressure and plastic deformation. The resulting debris may remain trapped at the interface, thereby promoting three-body abrasion and accelerating tool wear and surface deterioration. GI coatings, by contrast, are generally less susceptible to brittle fracture because of their greater deformability, but they are more likely to undergo smearing and adhesive transfer. This difference highlights that the dominant failure pathway is coating-dependent: GI sheets tend to exhibit adhesion-dominated damage, whereas GA sheets are more susceptible to fracture- and debris-assisted damage.

Along with friction and coating-damage tests, forming-limit evaluation is important for assessing the deformation capacity of galvanized steel sheets under complex strain paths. The forming limit diagram (FLD) describes the strain combinations at the onset of localized necking, while the forming limit curve (FLC) separates safe and failure regions under different major- and minor-strain conditions. Experimental FLCs are commonly determined using Nakajima-type hemispherical-punch tests or Marciniak-type tests in accordance with ISO 12004-2 and ASTM E2218. These tests are particularly relevant to galvanized sheets because the onset of localized deformation may coincide with, or precede, coating cracking, powdering, interfacial delamination, and surface damage in highly strained regions.

For GI sheets, the relatively soft and ductile Zn-rich coating can accommodate part of the substrate deformation; however, severe localized strain may still promote coating thinning, smearing, and adhesive transfer. By contrast, GA sheets contain brittle Fe–Zn intermetallic phases, and coating cracking or powdering may occur before the substrate reaches its macroscopic forming limit under certain bending, stretching, or frictional-contact conditions. Therefore, the conventional substrate-based FLC alone may not be sufficient to characterize coating integrity, especially for GA sheets. A coating-sensitive forming-limit assessment that combines the FLD/FLC with post-forming characterization of coating cracking, delamination, powdering, and surface roughness would be more suitable for evaluating the practical formability of GI and GA sheets.

At present, directly comparable experimental FLC datasets for GI and GA sheets under identical substrate grade, coating weight, lubrication condition, strain path, and testing procedure remain scarce. This lack of standardized data prevents a rigorous quantitative comparison of the intrinsic FLC differences between GI and GA coatings. Future studies should therefore establish coating-specific FLDs by combining standardized Nakajima or Marciniak tests with in situ strain measurement and post-test coating-damage characterization.

In summary, the failure behavior of GI and GA sheets during forming is controlled by the coupled action of adhesive wear, abrasive wear, and coating fracture. GI sheets are more susceptible to smearing, adhesion, and galling because of their softer Zn-rich outer layer, whereas GA sheets are more prone to cracking, delamination, powdering, and debris-induced abrasion because of the brittle nature of their Zn–Fe intermetallic coating. Under practical forming conditions, these mechanisms often interact and accelerate one another, leading to progressive surface degradation of both the sheet and the tool. Therefore, identifying the dominant failure mode and the conditions under which mechanism transitions occur is essential for understanding the tribological response of galvanized sheets and for optimizing tool design, lubrication, and forming parameters.

## 4. Governing Factors of Coating Failure

Process control plays a critical role in reducing frictional failure of GI and GA sheets. By optimizing lubricant selection and application methods in lubrication processes, improving die surface treatment techniques such as polishing and coating applications, and precisely regulating forming parameters including pressure, speed, and temperature, the coefficient of friction can be effectively reduced. This suppresses material adhesion and transfer, thereby mitigating various failure modes such as adhesive wear and abrasive wear. This approach represents a fundamental strategy for improving product reliability and economic efficiency from the manufacturing source. This section systematically reviews the effects of process parameters on the friction and wear behavior of galvanized sheets.

### 4.1. Tool Properties

Tool condition is a key external factor governing the forming behavior of galvanized steel sheets because it directly influences interfacial friction, coating damage, surface quality of stamped parts, and tool life [43,44]. For GA and GI coatings, differences in microstructure and mechanical properties lead to markedly different sensitivities to tool-related conditions [15,16,31,45]. Therefore, die hardness, die surface roughness, and die surface treatment or coating should be regarded as the principal parameters for evaluating and interpreting the forming performance of galvanized steel sheets. These factors not only affect frictional behavior at the sheet–tool interface, but also play a decisive role in coating failure, wear evolution, and the overall stability of the forming process [16,44,46]. From this perspective, the influence of tool condition on the forming behavior of GI and GA sheets can be systematically discussed in terms of die hardness, die roughness, and die surface treatment or coating.

#### 4.1.1. Die Hardness

The effect of die hardness on the surface integrity and tribological response of zinc-coated steels is governed by its influence on contact-stress distribution at the die–coating interface, the extent of plastic indentation, and the energy dissipated through shear-induced tearing. These effects operate differently in GI and GA steels because their coatings differ substantially in hardness, ductility, and microstructural uniformity.

**Figure 6:**
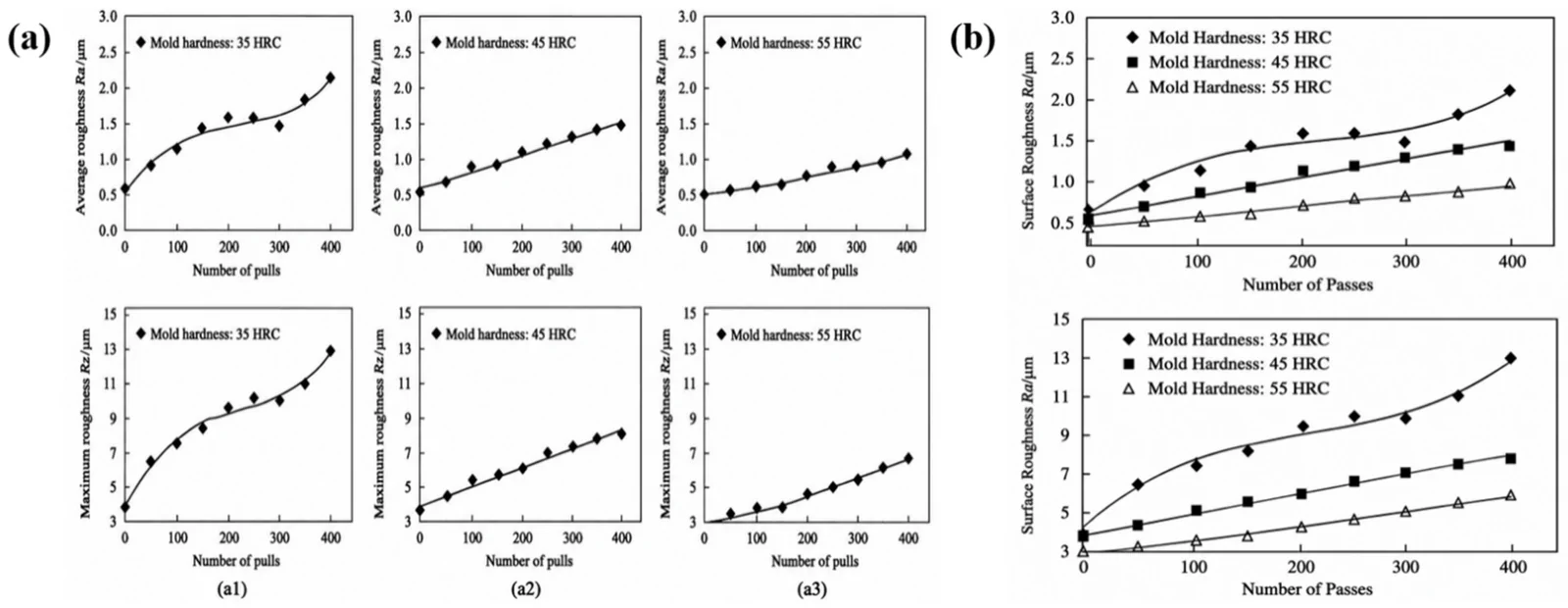
(**a**) Evolution of surface roughness and scratch depth of the galvanized steel sheet as a function of forming cycles under tools with nominal hardness values of (**a1**) 35 HRC (actual hardness: 33 HRC), (**a2**) 45 HRC, and (**a3**) 55 HRC (actual hardness: 54 HRC); (**b**) comparison of galling-induced surface deterioration of galvanized steel sheets formed with tools of different hardness [47].

As shown in Figure 6a, the surface roughness and scratch depth of the galvanized sheet increase progressively with the number of forming cycles for all tool hardness levels, confirming the cumulative nature of galling-related surface damage. However, the damage evolution is strongly dependent on tool hardness. With the 35 HRC tool (actual hardness: 33 HRC), the sheet roughness increased from approximately 0.6 to 2.2 μm after 400 cycles, while the scratch depth increased from about 4 to 13 μm. Increasing the tool hardness to 45 HRC substantially reduced the final roughness and scratch depth to approximately 1.5 μm and 8 μm, respectively. The 55 HRC tool (actual hardness: 54 HRC) produced the lowest damage level, with a final roughness of only about 1.0 μm and a scratch depth of approximately 6.5 μm. These results indicate that increasing die hardness suppresses progressive surface deterioration during repeated sliding and forming, most likely by improving the resistance of the tool surface to local plastic deformation, asperity damage, and zinc-assisted ploughing.

Figure 6b directly compares the effect of tool hardness on the evolution of galling damage. At an identical number of forming cycles, the 35 HRC tool consistently produces the highest sheet roughness and scratch depth, whereas the 55 HRC tool gives the lowest values throughout the test. The separation among the three curves becomes more pronounced with increasing cycle number, demonstrating that the benefit of higher tool hardness becomes increasingly important during prolonged forming. In particular, the difference between 35 and 55 HRC reaches approximately 1.2 μm in surface roughness and 6–7 μm in scratch depth after 400 cycles. Therefore, for the galvanized sheet examined in this study, higher die hardness improves resistance to galling-induced scratching and delays the accumulation of surface damage. Nevertheless, this monotonic trend should not be generalized directly to all galvanized coatings. While ductile GI coatings may benefit from a harder die surface, brittle GA coatings can exhibit an optimum intermediate hardness because excessively severe local contact stresses may promote cracking, powdering, and spallation of Fe–Zn intermetallic phases.

For GI steel, the influence of die hardness is generally monotonic, although a clear saturation behavior is often observed. Hou et al. [31] reported that, after 400 forming cycles, a die hardness of 52 HRC resulted in fewer scratches and lower surface damage than 35 HRC. Yu et al. [48] and Hou et al. [16] reported the same overall trend. Hou [45] using a comparable U-bend approach, further confirmed that GI surface deterioration decreases markedly as die hardness increases from 35 to 45 HRC, but shows little additional improvement at 52 HRC. This saturation behavior is consistent with the mechanical nature of the GI coating, which is relatively soft and ductile and can therefore act as a sacrificial lubricating interlayer between the tool and sheet [16,29,45]. As noted by Hou et al. [16], the GI coating has lower hardness, higher deformability, a smoother surface morphology, and a more uniform microstructure, all of which promote its lubricating role during sheet-metal forming. Hou et al. [16] further suggested that the soft zinc coating can accumulate in surface valleys and function as a solid lubricant. Consequently, increasing die hardness reduces plastic deformation of the die, suppresses mutual indentation and shear tearing of the zinc layer, and thereby lowers surface damage [16,31,45,48]. However, once the die hardness reaches approximately 45 HRC, the die becomes sufficiently resistant to ploughing by the coating, and further hardening no longer significantly reduces interfacial shear stress, leading to a plateau in performance [45,48].

By contrast, the dependence of GA steel on die hardness is non-monotonic, with an optimum at intermediate hardness. Previous studies showed that surface wear is severe at 35 HRC, decreases as die hardness increases to 45 HRC, and then rises again at 52 HRC after repeated forming cycles [16,45]. This trend has been consistently attributed to the brittle nature and heterogeneous microstructure of the GA coating [16,45]. Unlike GI, GA coatings are more prone to fragmentation, powdering, and spalling during deformation, and these failure modes become more pronounced when very hard dies impose contact stresses that exceed the cracking threshold of brittle coating phases. In particular, the brittle delta-1 phase has been reported to crack and exfoliate under excessively high contact stress, causing the tribological response to deteriorate rather than improve. EDS analysis also revealed a higher Fe content in GA coatings, which may intensify interfacial damage through stronger adhesion and mutual solubility between the sliding materials [16]. Yu et al. [15] likewise confirmed that excessive die hardness does not alleviate surface damage in GA steel and may even accelerate coating failure. Complementary strip-drawing results further indicate that, in GA systems, tribological performance may also depend on the die material’s resistance to ploughing by zinc debris and on the transition from ploughing-dominated to adhesion-dominated contact as die hardness increases. Therefore, unlike GI steel, GA steel does not benefit from continuously increasing die hardness; instead, an intermediate die hardness of 45 HRC provides more favorable balance between wear suppression and coating integrity in the studies summarized here [15,16,45].

It should be noted, however, that this GI/GA distinction may change when the substrate strength increases. For high-strength steel sheets, Yu et al. [48] found that 52 HRC produced substantially lower workpiece roughness than softer dies, and Hou et al. [7] proposed an empirical relationship in which friction or surface damage decreases with die hardness without an obvious saturation effect. Similar observations were reported by Szakaly et al. [49], who showed that harder tool steels generally reduce friction more effectively than cast iron across a wide range of pressure–velocity conditions. Nevertheless, hardness alone does not fully determine tribological behavior. Gaard et al. [50] demonstrated that a die material with a finer and more uniformly dispersed carbide population can maintain a less severe friction regime than a conventional hard tool steel under comparable loading conditions. This suggests that die microstructural uniformity must be considered together with bulk hardness when evaluating tool performance.

Collectively, the available evidence indicates that the influence of die hardness is coating-dependent. For GI steel, increasing die hardness is generally beneficial because the soft zinc coating acts as a lubricating and sacrificial interlayer, although the benefit becomes saturated once the die hardness reaches approximately 45 HRC. For GA steel, by contrast, excessive die hardness promotes coating cracking and spalling because of the brittle and heterogeneous nature of the coating; therefore, a moderate die hardness of 45 HRC is generally optimal. In practical terms, relatively high die hardness is recommended for GI-coated sheets, whereas GA-coated sheets require a controlled intermediate hardness window. In both cases, however, the final assessment should also account for substrate strength and die microstructural uniformity. The representative results reported in the literature are summarized in Table 3.

As shown in Table 3, the effect of die hardness differs markedly between GI and GA systems, not only in the severity of surface deterioration but also in the dominant failure mode. GI sheets generally exhibit progressive improvement in surface quality with increasing die hardness, whereas GA sheets tend to show an optimum at intermediate hardness, beyond which brittle coating damage becomes more pronounced.

#### 4.1.2. Die Roughness

Die surface roughness is one of the most influential parameters governing the tribological interaction between galvanized sheet and tool because it affects real contact area, lubricant retention, material transfer, and the initiation of coating damage during forming. Existing studies indicate that the effect of die roughness is not monotonic, but strongly depends on coating characteristics and die material [7,15,49,50,51].

For GI steel, Yu et al. [15] reported that a tool roughness of approximately 0.2 μm produced the least surface damage, whereas an excessively smooth tool surface (0.1 μm) was detrimental to sheet quality. This finding suggests that, for soft GI coatings, the optimum die roughness lies within a limited range rather than at the lowest-possible value. A moderately rough surface can retain zinc debris and lubricant within microvalleys, thereby promoting the formation of a stable tribofilm and reducing direct adhesion between the sheet and the die [15]. By contrast, both smoother and rougher dies can accelerate surface deterioration: the former by increasing intimate metal-to-metal contact and adhesive transfer, and the latter by enhancing ploughing and mechanical disruption of the coating.

For GA steel, the available evidence suggests a different trend. Yu et al. [15] reported that the surface roughness of the formed workpiece increased continuously with increasing tool roughness, indicating that smoother dies are generally more favorable for preserving GA surface quality. This behavior is consistent with the inherently harder and more brittle nature of GA coatings, in which die asperities are more likely to act as local stress concentrators, thereby promoting coating cracking, fragmentation, and spallation rather than the formation of a beneficial transfer layer [34]. However, direct studies on the effect of die roughness on GA steels remain limited, and the current understanding is still incomplete.

**Figure 7:**
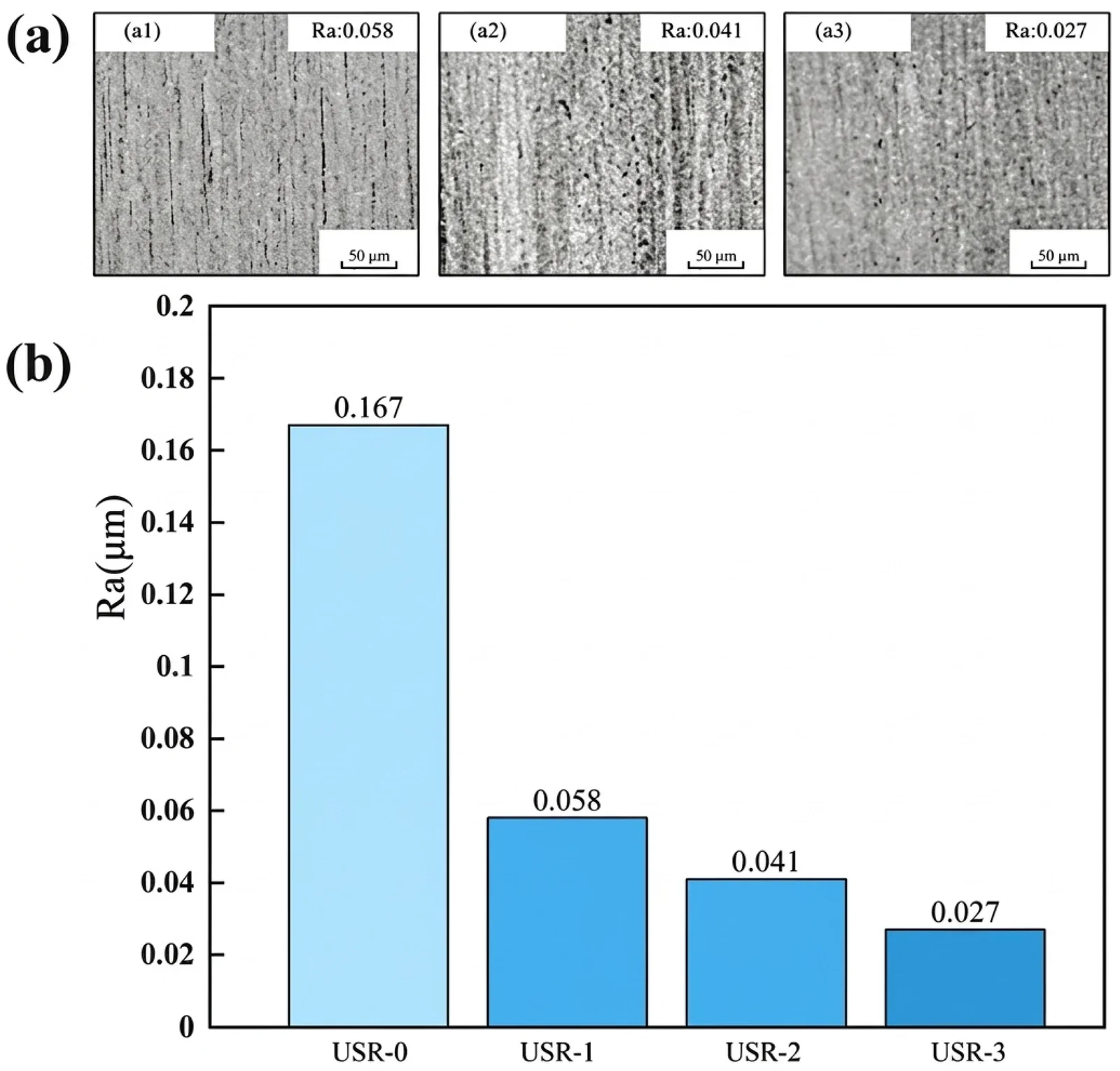
(**a**) Surface morphology of Cr12Mo1V1 die-steel components after ultrasonic impact numbers: (**a1**) USR-1 specimen, (**a2**) USR-2 specimen, and (**a3**) USR-3 specimen. (**b**) Roughness value of Cr12Mo1V1 die-steel components after ultrasonic impact numbers [52].

As illustrated in Figure 7, progressive refinement of the die surface significantly improves the surface quality of the formed galvanized sheet. The arithmetic mean roughness, Ra, decreases from 0.167 μm for the untreated surface (USR-0) to 0.058, 0.041, and 0.027 μm after successive surface-refinement stages (USR-1 to USR-3), corresponding to an overall reduction of approximately 84%. The corresponding optical micrographs show that the density and depth of longitudinal scratches gradually decrease as the surface roughness is reduced. At Ra = 0.058 μm, visible sliding marks and local defects remain apparent; these defects become less pronounced at Ra = 0.041 μm and are largely suppressed at Ra = 0.027 μm. These observations indicate that a smoother die surface can reduce asperity-induced ploughing and local stress concentration, thereby mitigating scratching and surface deterioration of the zinc coating. Nevertheless, for GI sheets, excessively smooth tools may reduce lubricant retention and increase the risk of direct adhesive contact. Therefore, die roughness should be optimized according to coating type and lubrication condition rather than minimized unconditionally [15,52].

Overall, current evidence suggests that GI sheets benefit from a narrow optimum die roughness window, typically around 0.15–0.25 μm, in which lubricant retention and zinc transfer help stabilize interfacial friction. By contrast, for GA sheets, lower die roughness appears generally more favorable because rough asperities tend to intensify brittle coating damage rather than assist in tribofilm formation. Nevertheless, the literature on GA-specific roughness effects remains limited, and further systematic studies are still needed to establish reliable roughness criteria for industrial forming applications.

#### 4.1.3. Surface Treatment

The effect of die surface treatment on the tribological behavior of GA and GI sheets is governed not only by the hardness of the deposited layer, but also by coating chemistry, post-treatment surface roughness, substrate load-bearing capacity, and the lubrication state at the die–sheet interface. Physical vapor deposition (PVD) and chemical vapor deposition (CVD) are widely used to enhance the surface properties of tool steels by producing hard and adherent nitride- or carbide-based coatings, such as Ti-, Cr-, Al-, and Zr-containing layers, which generally exhibit low friction, high oxidation resistance, and high hardness. However, the available evidence suggests that the tribological performance of these treated dies in GI and GA sheet forming cannot be interpreted on the basis of hardness alone, because the onset of scratching, galling, and coating damage depends on the combined evolution of interfacial adhesion, surface topography, and local contact stress [24,50,53,54,55].

Experimental studies on galvanized steels consistently show that die surface treatment can significantly reduce surface damage compared with uncoated tools, although the relative effectiveness of different coatings is strongly affected by surface condition. In ironing and stretching tests involving 40 sheets, Kim et al. [29] reported that uncoated tools exhibited severe scratching, whereas tools coated with PVD-CrN, PVD-XNP, and PVD-TiCN showed only localized coating removal near sharp tool radii. Although all coated tools had the same nominal hardness of 62 HRC, PVD-TiCN performed better than PVD-CrN and PVD-XNP, indicating that coating chemistry and coating–sheet interaction may be more important than hardness alone. However, interpretation of these results is complicated by the fact that the roughness values differed among the tested tools, ranging from 0.19 μm for the uncoated surface to 0.35 μm for the PVD-TiCN-coated surface. Since roughness affects local stress concentration, lubricant retention, and the likelihood of coating fracture or zinc transfer, it acts as a major confounding factor in evaluating coating performance [24,51,55]. Therefore, the available data indicate that PVD coatings do exert a beneficial effect on the formability of GI/GA sheets. However, the relative performance ranking of different coating types cannot be reliably determined without strict control over surface roughness.

A comparable conclusion was reached by Altan et al. [30], who evaluated different tool and coating materials using strip-drawing and ironing tests under high contact pressure. Their results showed that, after 40 consecutive tests on GI steel, DC53 tools coated with TiCN and K340 tools coated with PVD-CrN exhibited the lowest wear. Nevertheless, because the study did not separately account for differences in substrate hardness and surface roughness, the observed performance differences cannot be unambiguously attributed to the coatings themselves. This limitation is important because both the load-bearing capacity of the substrate and the topographical state of the coated surface affect whether the coating can remain intact under repeated sliding [53,54]. Eriksson and Olsson [53] for example, demonstrated that coating behavior depends strongly on both coating chemistry and the substrate-coating support system: carbon-based CrC/C coatings showed consistently low material transfer against hot-rolled, cold-rolled, and zinc-coated high-strength steels, whereas CrN was more susceptible to Zn pickup on galvanized sheets. They further noted that reduced substrate hardness lowers the mechanical support of the coating and increases the tendency for brittle coatings to crack and spall. These observations indicate that, in galvanized sheet forming, effective die surface treatment requires not only a suitable coating material but also sufficient substrate support to prevent premature coating failure.

**Figure 8:**
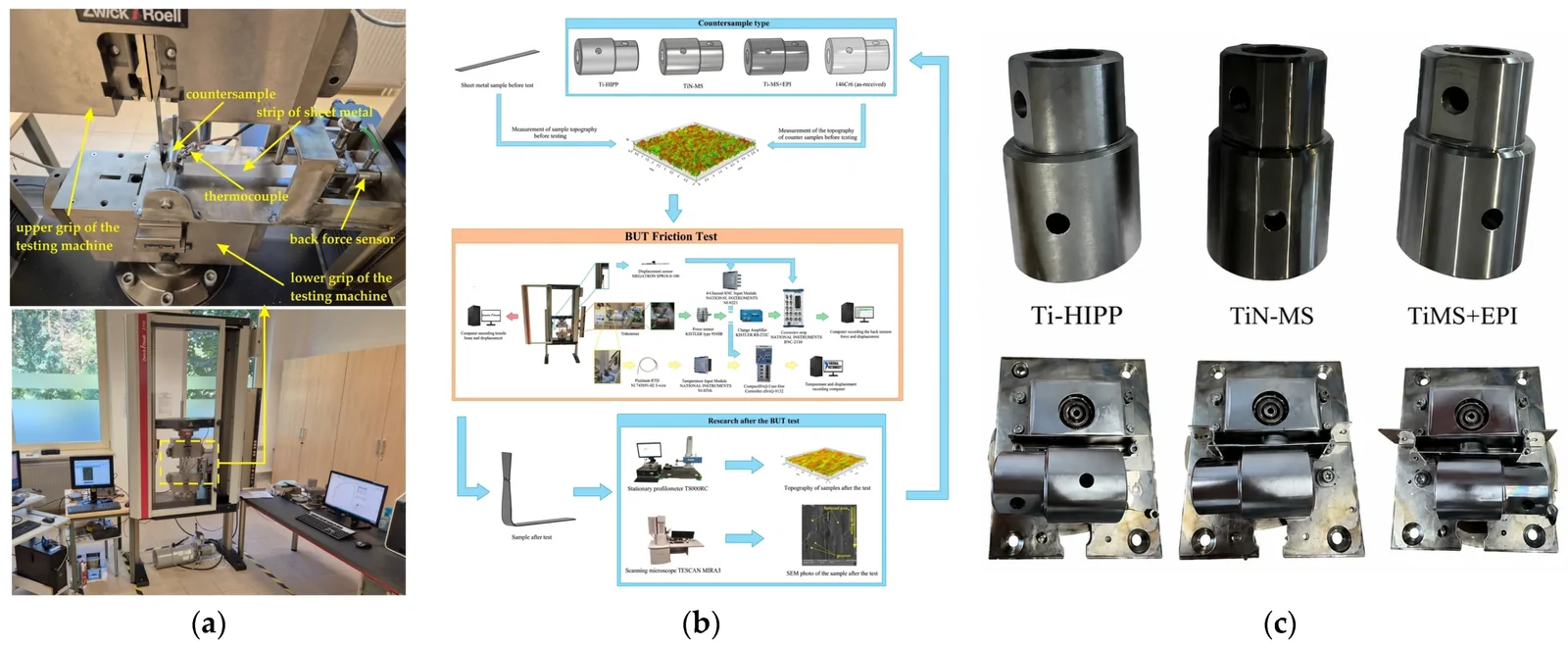
(**a**) BUT test stand; (**b**) research route for investigating the influence of various titanium and titanium nitride coatings on the surface of 145Cr6 cold-work tool steel samples; (**c**) general view of the modified countersamples, namely Ti-HIPP, TiN-MS, and TiMS + EPI, together with three consecutive orientations of the countersample during electron-gun modification [55].

In addition to PVD and CVD coatings, other surface engineering methods have also been shown to improve resistance to wear and galling by modifying both near-surface hardness and interfacial affinity. Wang et al. [54] reported that nitriding of DC53 tool steel formed an Fe3N/Fe4N composite layer that increased near-surface hardness and delayed the onset of galling, whereas thermal diffusion treatment generated a hard VC layer with strong metallurgical bonding and lower chemical affinity to the workpiece, thereby providing the best overall galling resistance. As shown in Figure 8, Trzepieciński et al. [55] similarly showed in bending-under-tension tests that protective surface treatments stabilize the friction coefficient during sliding and reduce the rise in contact-zone temperature compared with uncoated surfaces, indicating that such treatments can also suppress frictional heating and the associated surface degradation. For galvanized advanced high-strength steels, Kim et al. [29] further demonstrated that the onset of powdering and galling is governed primarily by contact pressure and tool roughness rather than by flash temperature, emphasizing that interface geometry and contact state remain central to the success of any coating system. Taken together, these results suggest that die surface treatment improves GI and GA sheet forming not simply by increasing hardness, but by reducing adhesion, stabilizing friction, and delaying the evolution of damage at the contact interface [10,24,55].

The available literature further indicates that coating selection should be matched to the workpiece material rather than chosen according to a single hardness criterion. Podgornik et al. [51] showed that low-friction carbon-based coatings such as WC/C and DLC can minimize adhesive transfer more effectively than harder nitride coatings in adhesion-prone systems, particularly under poor lubrication. Sato et al. [56] likewise reported that DLC-coated tools significantly reduced wear and galling in sheet aluminium forming, even under mild lubrication or dry conditions. However, Podgornik et al. [57] emphasized that nitride coatings or nitrided surfaces may be more effective for some workpiece materials, confirming that no universally optimal coating system exists. This conclusion is also relevant to GI and GA sheets, for which interaction with the zinc-based surface layer introduces additional complexity. In particular, post-deposition polishing has been shown to markedly reduce friction and delay material transfer by removing asperities introduced during coating deposition [37,51]. For galvanized sheets, such asperities may otherwise act as preferred sites for Zn accumulation, local coating fracture, or galling initiation. Consequently, the final tribological performance of a treated die is determined not only by the intrinsic properties of the coating but also by the surface morphology retained after deposition and finishing [37,51].

Based on the available evidence, the effect of die surface treatment on GA and GI sheet forming is best understood as a coupled surface-engineering problem involving coating chemistry, roughness control, substrate support, and lubrication compatibility. Coated and surface-treated dies generally outperform uncoated tools by reducing scratching, friction instability, and galling [29,30,54]. However, the available evidence also shows that higher coating hardness alone does not guarantee better performance. Instead, the most effective die surface treatments are those that combine low interfacial adhesion, good coating integrity, adequate load-bearing support, and a smooth post-treatment surface condition [30,37]. Therefore, for GI and GA sheet forming, die surface engineering should be designed according to a combined criterion of low roughness, strong coating–substrate bonding, sufficient mechanical support, and low chemical affinity to zinc, rather than a simple pursuit of harder coatings.

### 4.2. Lubrication Conditions

Lubrication conditions are among the most important factors governing the frictional behavior, coating damage, and zinc powdering tendency of galvanized steel sheets during stamping. Existing studies have mainly focused on lubricant type, lubricant viscosity, oil-film thickness, or oil amount, and the development of novel lubricant films or surface lubricating coatings. In general, an appropriately designed lubrication system can reduce the friction coefficient and forming load at the tool–sheet interface, minimize direct contact between the die and the zinc coating, and thereby suppress coating scratching, adhesive wear, and zinc debris generation. However, the influence of lubrication parameters is not simply monotonic; rather, it depends strongly on coating type, surface morphology, contact pressure, sliding speed, temperature, and additive compatibility [32,58,59,60,61,62]. From this perspective, lubrication should be understood not merely as a means of friction reduction, but as a critical factor controlling interfacial separation, debris evolution, and the transition between different coating-failure modes.

#### 4.2.1. Lubricant Type

Lubricant type is the primary factor determining the interfacial friction state of galvanized steel sheets. Because different lubricants vary in film-forming ability, load-carrying capacity, chemical affinity, and compatibility with zinc-coated surfaces, they can markedly affect the friction coefficient, punch force, coating wear, and powdering tendency. Sener et al. [63] compared polyvinyl chloride (PVC), polyethylene, and polytetrafluoroethylene films with mineral oil by means of cupping tests combined with finite element analysis, and found that all lubricants reduced interfacial friction and punch force during stamping of galvanized steel sheets. The punch force–stroke curves under different lubrication conditions indicate that the PVC film provided the best overall lubricating performance. Gong et al. [64] further investigated the interfacial friction behavior during deep drawing of hot-dip galvanized sheets using a probe-based measurement method and reported substantial differences among lubricants. Among conventional lubricants, PK oil showed relatively favorable performance; after the addition of solid additives, the newly developed L-series lubricants further improved the frictional condition, with LB oil outperforming LA oil. These results indicate that both the base lubricant and the additive formulation play important roles in friction reduction and suppression of coating damage.

In addition to the base lubricant, the compatibility between additives and the galvanized surface is also critical. Schey and Dalton [32] showed that forming of galvanized materials generally occurs under mixed lubrication conditions and that an appropriate lubricant can reduce the boundary-contact area and surface scratching. However, the effect of additives is not always beneficial. Chlorine-containing additives generally help reduce friction and scratching, whereas sulfur- and phosphorus-containing additives may, under certain conditions, increase friction or aggravate surface damage. This suggests that insufficient compatibility between the lubricant system and the zinc coating may promote coating abrasion, accumulation of zinc debris, and powdering. Subramonian et al. [65], based on strip-drawing tests, deep-drawing tests, and numerical simulations, reported that water-based and synthetic lubricants generally outperform conventional petroleum-based lubricants. These lubricants not only reduce the interfacial friction coefficient, but also improve the forming limit and enhance forming stability under high blank-holder forces. Lanzon et al. [66] further found that conventional rolling oils tend to fail under high contact pressure because of their limited load-carrying capacity, leading to oil-film rupture and (stick slip action) stick–slip behavior. This in turn causes repeated contact and seizure between the die and the coating, thereby intensifying coating spallation and zinc powder generation. Bay et al. [61] also pointed out that, in severe friction regions such as draw beads, additional lubrication may reduce friction but may also facilitate separation and transfer of worn zinc particles to the tool surface, thereby aggravating galling and adhesion.

Overall, the effect of lubricant type can be understood from three interrelated aspects: reduction of the friction coefficient and forming load, suppression of direct die–coating contact and coating wear, and influence on the generation, transport, and re-adhesion of zinc debris through tribochemical interactions. Therefore, optimization of lubricant type should consider not only friction reduction, but also its effects on zinc particle generation, transfer, and accumulation at the interface. From Schey’s bead drawing test results, it can be seen that the optimal system for both types of sheet materials in the laboratory is 285 mm^2^/s high-viscosity paraffin base oil combined with 5% chlorinated paraffin. This system relies on a high oil-film ratio to reduce boundary contact, and chlorine-based additives can inhibit the peeling and sticking of the GI zinc layer, and is suitable for the oil storage characteristics of GA porous coatings. Under chlorine-free light load conditions, high-viscosity oil can be used in combination with 1% stearic acid. Sulfur and phosphorus extreme pressure agents are not suitable for GI and may exacerbate surface scratches. The Erichsen cupping test confirmed that PVC film is the optimal solid lubricant material for both GI and GA, with the highest molding limit and the lowest impact pressure. For industrial production that meets REACH environmental requirements, GA prioritizes the use of high-viscosity synthetic esters + low phosphorus, fatty acid chlorine-free lubricants, while GI requires the use of plant-based/synthetic ester lubricants in conjunction with TiAlN/DLC hard-mold coatings. Pre-coated polymer dry films on coils can be used as a universal cleaning solution for both types of sheets, and low-viscosity base oils and pure mineral oils are not suitable for stamping galvanized sheets.

#### 4.2.2. Lubricant Viscosity

Lubricant viscosity is an important parameter controlling oil-film load-carrying capacity and interfacial contact conditions. In general, higher viscosity is more favorable for forming a thicker and more stable lubricant film between the die and the sheet, thereby reducing direct asperity contact and lowering friction, scratching, and coating delamination. Schey and Dalton [32] found that increasing the viscosity of paraffinic mineral oils increased oil-film thickness, reduced the boundary-contact area, and alleviated scratching and coating scuffing on galvanized sheets. Schey [59] further reported that forming of galvanized sheets is mainly governed by mixed lubrication and that the friction coefficient decreases with increasing values of the viscosity–speed parameter, indicating that increasing lubricant viscosity or forming speed within a certain range can enhance hydrodynamic effects and reduce adhesive transfer during the initial contact stage.

Lee et al. [62] investigated the influence of lubricant viscosity and surface roughness on the friction behavior of various zinc-based coated sheets, and showed that lower lubricant viscosity leads to a higher friction coefficient, whereas higher viscosity helps maintain a more continuous lubricant film and reduces direct die–sheet contact. Li et al. [67] similarly reported for galvannealed steel sheets that the friction coefficient decreases with increasing lubricant viscosity, while higher-viscosity lubricants and appropriate forming speeds contribute to more stable lubrication and less coating wear and zinc powdering. However, under high-temperature and high-pressure conditions, lubricant film failure becomes more likely, resulting in severe adhesion and tearing of the coating. From a tribological perspective, Lee et al. [68] emphasized that lubricant viscosity and oil-film integrity directly determine the interfacial contact state of galvanized sheets. Under boundary and mixed lubrication conditions, the zinc coating is more susceptible to adhesive wear, whereas selecting a lubricant with suitable viscosity and maintaining a stable oil film are essential for reducing coating spallation and zinc powder formation.

Therefore, the effect of lubricant viscosity on zinc powdering can be summarized as follows: when viscosity is too low, the load-carrying capacity of the oil film is insufficient, leading to increased direct contact between the die and the zinc coating and thus promoting adhesive wear and zinc-particle detachment. A moderate increase in viscosity can enhance oil-film stability and reduce friction as well as coating damage. However, viscosity selection should still take into account temperature, forming speed, contact pressure, and post-forming cleaning requirements, so as to avoid non-uniform application or reduced process compatibility caused by excessively high viscosity.

#### 4.2.3. Film Thickness and Oil Amount

Oil-film thickness, or the amount of lubricant per unit area, directly determines whether the lubricant can adequately cover the zinc-coated surface and provide effective separation in asperity contact regions. When the oil-film is too thin or local starvation occurs, the lubrication regime readily shifts toward boundary lubrication, causing adhesive interaction, ploughing, and coating delamination. By contrast, once the oil amount exceeds a certain level, the benefit in friction reduction tends to saturate, and excessive oil may even lead to non-uniform lubrication because of oil migration or squeeze-out. Rangarajan [69] showed that increasing the amount of pre-applied oil improves the formability of galvannealed sheets, but when the oil amount reaches approximately 3 g/m^2^, further increase brings little additional benefit. Moreover, oil migration during storage may cause local non-uniformity in oil distribution, indicating that an appropriate oil amount is more important than simply maximizing lubricant application.

**Figure 9:**
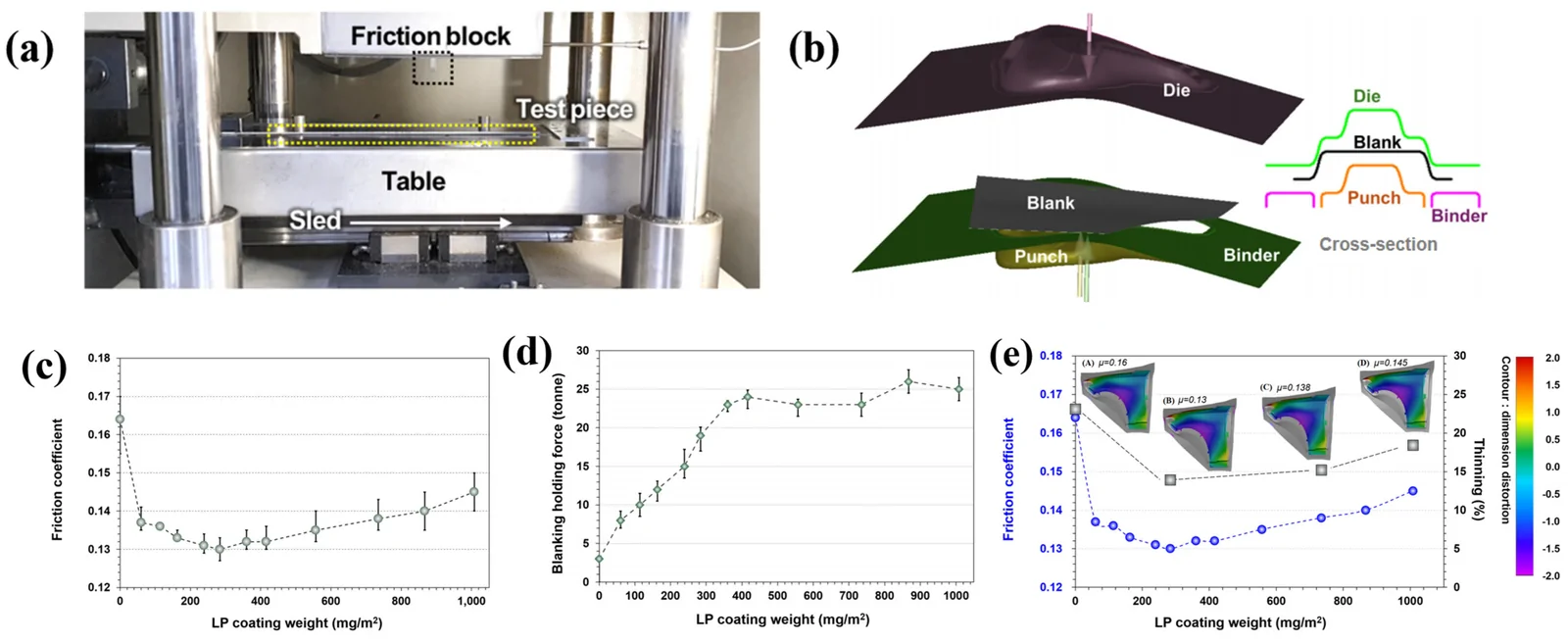
(**a**) Single-sided sliding-type friction test apparatus used for friction coefficient measurements. (**b**) Stamping simulation model using experimentally measured friction coefficients as input parameters. (**c**) Variation in friction coefficient as a function of LP coating weight. (**d**) Variation in cup-drawing limit blank-holding force as a function of LP coating weight. (**e**) Integrated relationship among LP coating weight, friction coefficient, thickness reduction, and dimension distortion [70].

As shown in Figure 9, Kim et al. [70] confirmed that the amount of oil applied significantly changes the stamping friction and forming ability of GA sheet metal: the friction coefficient is highest when there is no oil/low oil amount, the ultimate edge pressure of cup protrusion is only three tons, and the local thinning of the parts is severe. The oil application rate has increased to 200–300 mg/m^2^, and the friction coefficient has dropped to a minimum of 0.130. The material flows smoothly, the ultimate edge pressure has been significantly increased, and the stamping thinning has been significantly alleviated. When the oil coating exceeds 550 mg/m^2^ and enters the high oil range, the uneven lubrication film causes the friction coefficient to rise to 0.145, and the forming improvement effect tends to saturate. Finite element simulation also confirms that the friction coefficient directly determines the degree of local thinning of the component, and the size rebound is weakly affected by the oil coating. Miguel et al. [71] studied the friction behavior of electrogalvanized low-carbon steel sheets under pre-applied mineral-oil conditions and found that differences in residual oil caused by different draining times had no significant effect on the friction coefficient. This suggests that, in some pre-lubricated systems, the initial oil-film thickness is not the sole controlling factor and that contact pressure also plays an important role in determining the effective lubrication state. Zhou et al. [72], through flat sliding tests, reported that within a certain range the friction coefficient gradually decreases with increasing oil-film thickness and sliding speed, and tends to stabilize once the oil-film thickness exceeds about 6 μm, indicating the existence of an effective lubrication range. Dalton and Zaccone [60] further found that galvannealed sheets are more sensitive to oil amount than electrogalvanized sheets; under low-oil conditions, the friction coefficient of galvannealed sheets increases markedly. By contrast, electrogalvanized sheets are less sensitive to oil amount but more sensitive to lubricant type, suggesting that different zinc-coated surfaces respond differently to lubricant quantity and chemistry. Coello et al. [73] also pointed out that oil-film thickness has little effect on the friction coefficient under low-speed conditions, whereas at higher speeds a thinner film leads to a slight increase in friction. In addition, interactions between the oil film and surface microtexture may generate microhydrodynamic pressure, thereby improving friction and wear performance.

Taken together, the effect of oil-film thickness and oil amount exhibits a clear interval-dependent characteristic: insufficient lubrication increases boundary contact and adhesive wear, moderate oil addition helps stabilize lubrication, whereas further thickening beyond the stable range brings only limited additional benefit. This indicates that the objective of oil application is not simply to maximize oil quantity, but to maintain a sufficiently stable and spatially uniform lubricant film throughout forming.

#### 4.2.4. Novel Lubricant Films and Lubricating Coatings

With increasing surface-quality requirements for automotive galvanized steel sheets, conventional liquid lubricants alone are sometimes insufficient to meet the demands of complex forming operations in terms of friction reduction and powdering suppression. Consequently, recent studies have increasingly focused on solid-film lubricants, dry-film lubricants, and inorganic lubricating coatings as alternative strategies for interfacial control. Schey [58] investigated the effects of several solid-film lubricants, including paraffin, microcrystalline wax, stearic acid, glyceryl tristearate, and sodium stearate, on the forming-friction behavior of different galvanized sheets using draw-bead tests. The results showed that the effectiveness of solid-film lubrication depends not only on the lubricant itself but also on the surface morphology and hardness of the coating. Overall, microcrystalline wax and paraffin performed relatively well, whereas some fatty-acid-based films may fail locally because of insufficient film retention.

In terms of functional surface coatings, Yoshiharu et al. [74] prepared an approximately 50 nm Ni–Fe–O ultrathin inorganic lubricating layer on galvannealed steel sheets, which significantly reduced the friction coefficient and drawing force and enabled forming performance comparable to that of duplex galvannealed sheets. This improvement was attributed to enhanced anti-adhesion properties of the modified surface and improved affinity for stamping oils and extreme-pressure additives, thereby lowering the shear resistance in local metal-to-metal contact zones. Higai et al. [75] developed a composite organic dry-film lubricant that effectively reduced interfacial friction and suppressed coating flaking and zinc powder generation during stamping, while still maintaining acceptable compatibility with alkaline degreasing, phosphating, and spot welding. Ochiai et al. [76] developed a nanoscale Mn–P inorganic lubricating coating that combines an Mn-rich anti-adhesion layer with a P-rich friction-reducing layer acting synergistically with lubricating oil, thereby significantly suppressing die pickup and coating delamination. Kawasaki Steel [77] also developed an inorganic dry-film-lubricated galvannealed steel sheet with good friction-reduction capability, anti-adhesion performance, and process compatibility. These studies demonstrate that novel lubricant films and surface lubricating coatings can further suppress coating delamination and zinc powder generation through interfacial separation, anti-adhesion effects, and synergistic friction reduction, and therefore represent an important future direction for powdering control in galvanized sheet stamping.

Overall, existing studies have systematically demonstrated the strong influence of lubrication conditions on the tribological behavior and zinc powdering tendency of galvanized steel sheets. In general, water-based and synthetic lubricants, as well as specially formulated drawing oils containing extreme-pressure additives, usually exhibit better friction-reducing and anti-adhesion performance than conventional rolling oils. Appropriate increases in lubricant viscosity and proper control of oil-film thickness can improve load-carrying capacity, maintain a stable lubrication state, and reduce coating damage. In addition, solid films, organic dry films, and inorganic lubricating coatings provide promising new approaches for suppressing coating delamination and zinc debris generation by improving interfacial separation and anti-adhesion behavior. Nevertheless, lubrication performance remains strongly coating-dependent, and the interactions among lubricant chemistry, coating microstructure, contact pressure, sliding speed, and surface topography still require more systematic investigation.

### 4.3. Forming Parameters

Forming conditions play a decisive role in the tribological response and coating-failure behavior of galvanized steel sheets because they directly determine local contact pressure, deformation path, sliding condition, and thermal state at the sheet–tool interface. In practical stamping, variables such as blank-holder force, normal pressure, forming speed, and temperature do not act independently; rather, they interact to influence lubrication breakdown, coating fracture, adhesion, powdering, and final surface quality. Therefore, understanding the role of forming conditions is essential for interpreting coating damage and optimizing process reliability.

#### 4.3.1. Blank-Holder Force

Blank-holder force is a key process parameter in sheet-metal stamping because it governs material flow in the flange region and directly affects contact pressure, frictional shear, and coating damage at the sheet–tool interface. Aleksandrović and Stefanović [78] pointed out that blank-holder force essentially represents the normal load applied to the flange during frictional restraint: if it is too low, wrinkling and unstable material flow may occur, whereas if it is too high, frictional resistance increases, the thickness of the critical zone decreases, and the sheet becomes more prone to fracture. For galvanized steel sheets, this balance is particularly important because sliding contact between the zinc coating and the die can promote coating removal and transfer to the tool surface, thereby progressively worsening tribological conditions. In this context, insufficient blank-holder force may induce abnormal local contact through wrinkling, whereas excessive blank-holder force may intensify zinc powdering, coating peeling, and die sticking owing to elevated contact pressure and interfacial shear.

**Figure 10:**
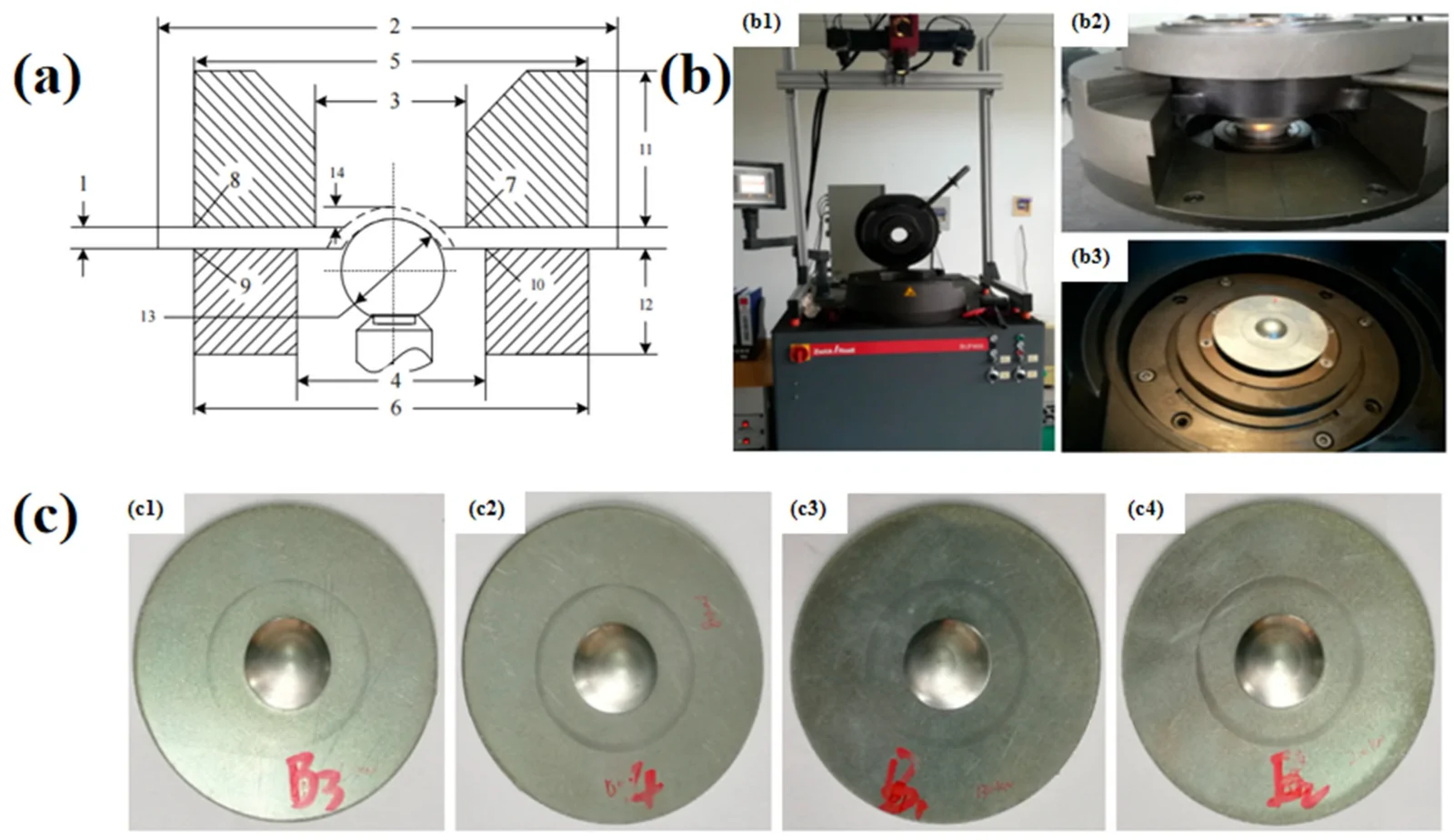

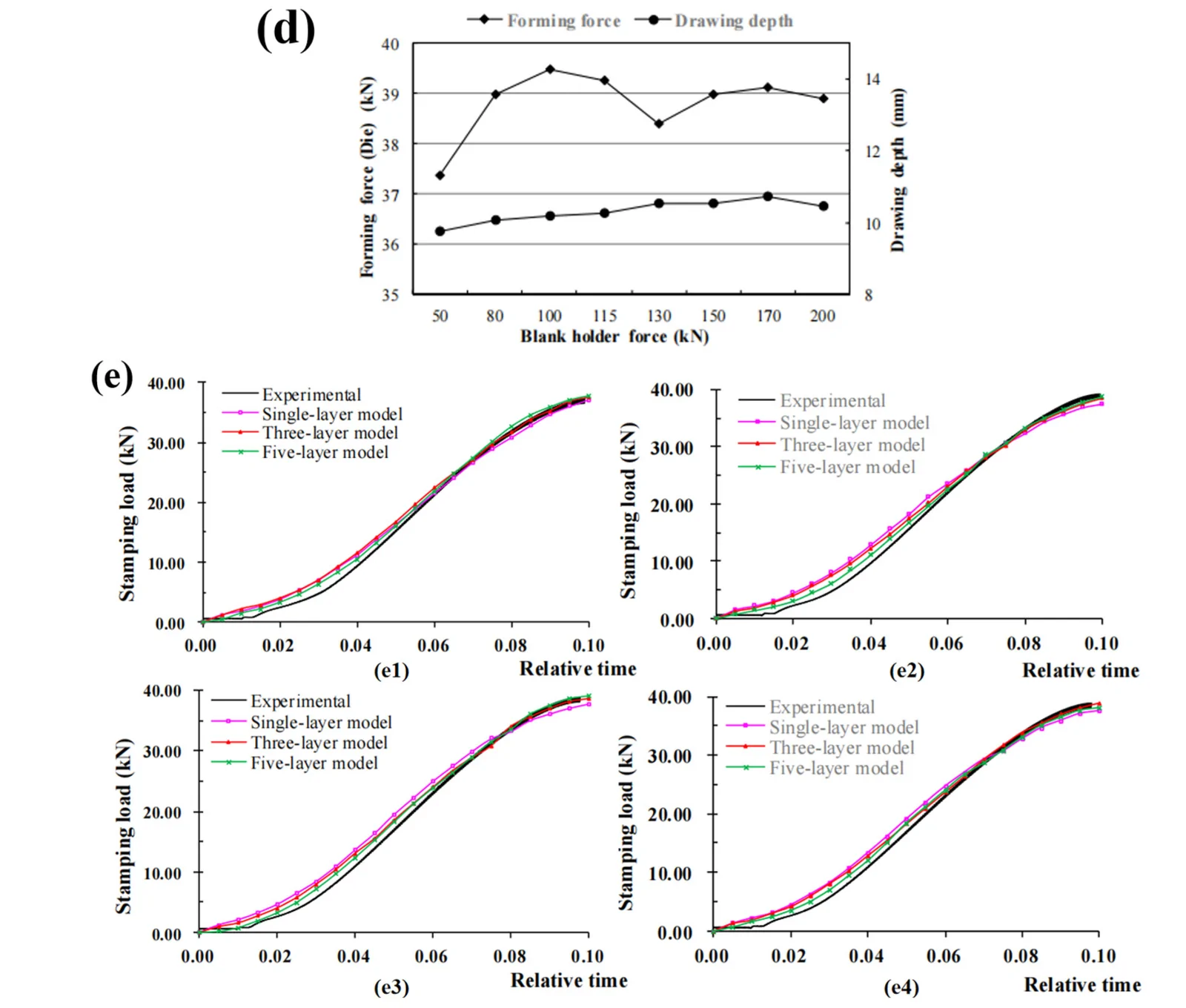
(**a**) Hemispherical part drawing model (mm). (**b**) Hemispherical part drawing test device: (**b1**) BUT400 drawing machine, (**b2**) hemispherical part drawing die, (**b3**) Lower die of hemispherical part drawing die. (**c**) Part samples of hemispherical part drawing test under different blank holder force: (**c1**) Sample of 50 kN test, (**c2**) Sample of 80 kN test, (**c3**) Sample of 130 kN test, (**c4**) Sample of 200 kN test. (**d**) Variation of forming force and drawing depth under different blank holder forces. (**e**) Stamping force history comparative between numerical simulation results of different element models and experimental results: (**e1**) Blank holder force 50 kN, punch stroke 9.76 mm, (**e2**) Blank holder force 115 kN, punch stroke 10.25 mm, (**e3**) Blank holder force 170 kN, punch stroke 10.75 mm, (**e4**) Blank holder force 200 kN, punch stroke 10.45 mm [81].

This trend is supported by several studies. Fu et al. [79] showed through finite element simulation of rectangular-box deep drawing that the blank-holder-force path significantly affects maximum thinning and forming quality, implying that unreasonable force conditions may indirectly aggravate coating cracking and detachment through strain localization and frictional intensification. Shisode et al. [80], in cupping and deep-drawing tests on DX54 hot-dip galvanized steel, further demonstrated that low blank-holder force (10 kN) promoted flange wrinkling and altered the actual contact state, making conventional simulations less accurate, whereas higher forces (30 and 50 kN) effectively suppressed wrinkling and improved agreement between experiment and simulation. As shown in Figure 10, Li and Long [81] also reported, based on hemispherical drawing tests and cohesive-zone finite element modeling of galvanized DP600, that when the edge pressure of the workpiece gradually increases from 50 kN to 170 kN, the ultimate stamping depth continues to increase; however, once the edge pressure of the workpiece exceeds 170 kN, the ultimate stamping depth begins to decrease. The forming force shows an overall upward trend with the increase of the workpiece edge pressing force: when the workpiece edge pressing force is insufficient, the material flow in the flange area cannot be effectively controlled; excessive edge pressure on the workpiece can increase the frictional resistance of the sheet metal, leading to premature fracture of the workpiece and reducing the forming limit. In addition, Raab et al. [82] compared conventional hot-dip galvanized sheet (HDG/Z) and ZnAlMg-coated sheet (HDG/ZM) under blank-holder forces of 20–240 kN and found that HDG/ZM exhibited a lower friction coefficient, greater limiting drawing depth, and better resistance to coating peeling and die sticking, particularly under high-pressure conditions.

Overall, current studies indicate that blank-holder force exerts a dual influence on zinc powdering during galvanized sheet stamping: excessively low force leads to unstable flow and wrinkling, whereas excessively high force increases contact pressure, friction, and local thinning, thereby promoting coating cracking, powdering, and delamination. Therefore, blank-holder force should be optimized together with lubrication, draw-bead design, and die geometry in order to minimize coating damage and improve forming stability.

#### 4.3.2. Normal Pressure

Normal contact pressure is another critical factor influencing zinc powdering during stamping of galvanized steel sheets because it directly modifies the interfacial contact state, real contact area, and frictional shear between the coating and the die. Evin et al. [83] noted that the friction and wear behavior of Fe–Zn-coated steels is governed not only by external load, but also by lubrication, surface microstructure, and drawing speed. As normal pressure increases, die asperities are more likely to penetrate the coating surface, thereby intensifying coating damage and abrasive-particle detachment.

**Figure 11:**
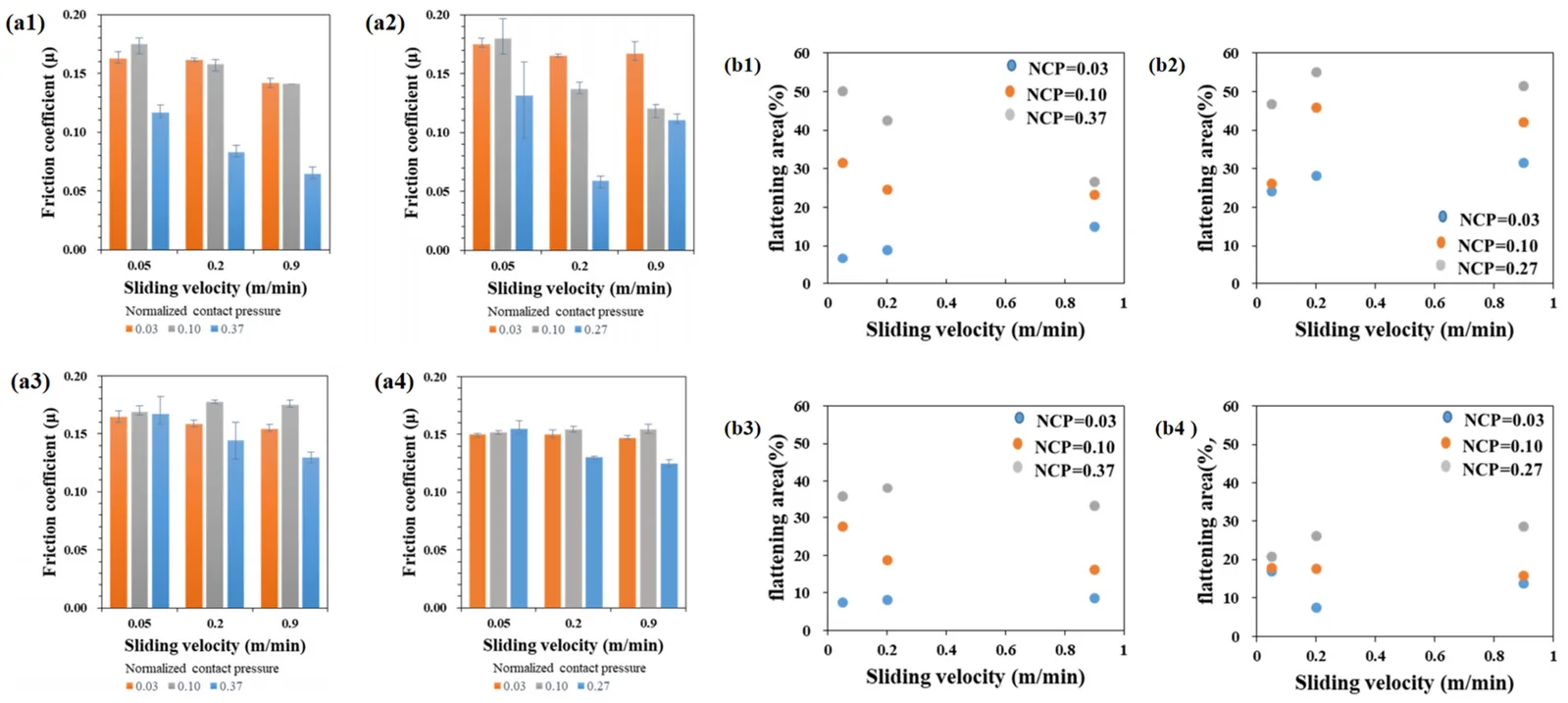
Variation in the measured friction coefficient with sliding velocity and normalized contact pressure for (**a1**) TS 340-GI, (**a2**) TS 980-GI, (**a3**) TS 340-GA, and (**a4**) TS 980-GA. The corresponding percentage of flattened area calculated as a function of sliding velocity and normalized contact pressure is shown in (**b1**)–(**b4**) for (**b1**) TS 340-GI, (**b2**) TS 980-GI, (**b3**) TS 340-GA, and (**b4**) TS 980-GA [10].

As shown in Figure 11, This effect was further clarified by Kim et al. [10], who showed in friction tests on GI and GA sheets under different pressures and sliding speeds that contact pressure strongly affects asperity flattening and frictional response. Because GI coatings are softer, they are more prone to plastic flattening under load, whereas GA coatings, which contain hard and brittle Fe–Zn intermetallic phases, are more susceptible to powdering damage under the combined action of high normal pressure and sliding shear. Similar conclusions were reported by Reinberg et al. [84], who found that galling and powdering increased with contact pressure, whereas lubrication significantly delayed the onset of these failures. This explains why regions subjected to high local pressure, such as draw beads, die radii, and compression zones, are often the most vulnerable to zinc transfer and die sticking in industrial stamping. Park et al. [85] further distinguished between powdering and flaking in GA coatings, suggesting that powdering is mainly associated with fracture of brittle phases within the coating, whereas flaking is more closely related to failure at the coating–substrate interface. This indicates that contact pressure may influence not only the severity but also the dominant mode of coating failure. Peng et al. [86] also showed in their study of hot-stamping wear of galvanized ultra-high-strength steel that coating loss is closely related to the actual contact area, which increases with normal pressure and enhances interfacial shearing and ploughing.

The available evidence indicates that excessive normal pressure promotes zinc powdering by increasing real contact area, weakening lubrication, intensifying frictional shear, and accelerating coating fracture, delamination, and adhesive transfer. Therefore, controlling local contact pressure through appropriate blank-holder force, draw-bead design, die-radius optimization, lubrication, and die-surface engineering is essential for reducing coating damage during galvanized sheet stamping.

#### 4.3.3. Speed

Stamping speed is another important process parameter affecting the forming behavior of galvanized steel sheets because it alters the tribological state at the sheet–tool interface and thereby influences friction, coating transfer, zinc powdering, and final part quality. Schey [59] showed, using draw-bead simulation tests on galvannealed, electrogalvanized, and hot-dip galvanized sheets, that the friction coefficient generally decreased with increasing combined effects of drawing speed and lubricant viscosity. However, under low-speed conditions, some electrogalvanized and hot-dip galvanized sheets exhibited frictional instability, coating transfer, and surface damage. Consistent with this, Sanchez [87] reported from stretching and clamping tests that electrogalvanized sheets showed more severe coating adhesion to the die at low speed, which could increase friction and even promote sheet fracture.

**Figure 12:**
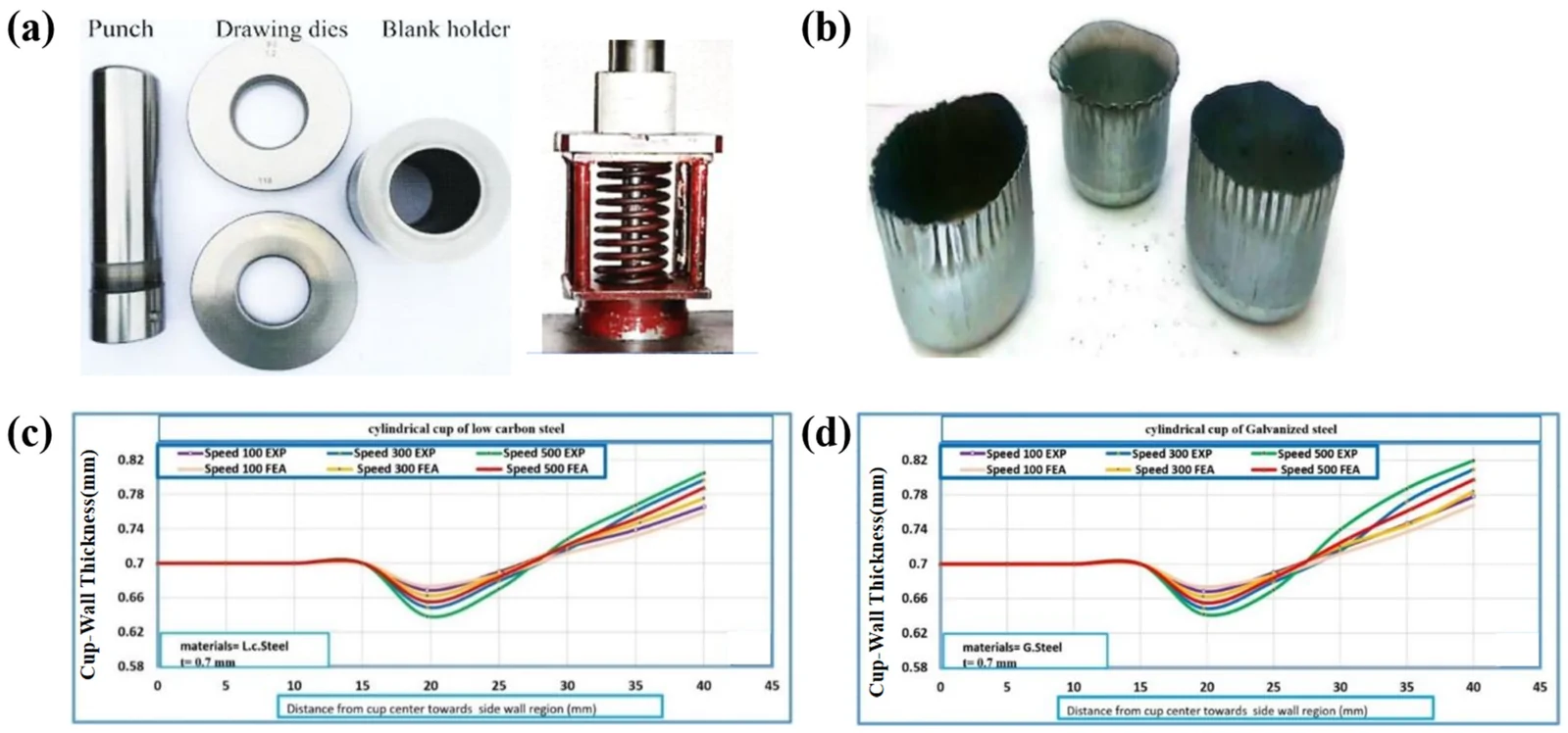
(**a**) Cylindrical rollers, punches, and dies used in the production process; shows the deep drawing die employed. (**b**) The cup edge is in a failed state. (**c**) Thickness distribution of low-carbon steel at different stamping speeds. (**d**) Thickness distribution of galvanized steel at different punching speeds [91].

Kim and Lee [88] further demonstrated in cup-forming experiments that punch speed affects punch load and strain distribution through its influence on friction, while Keum et al. [89] emphasized that the speed dependence of friction should be incorporated into finite element models of galvanized sheet forming. Similar conclusions were reached by Yim et al. [90], who noted that friction variations with forming speed are important for accurate formability prediction in automotive galvanized steels. As shown in Figure 12, although Abdullah et al. [91] found that increasing punch speed aggravated local thinning during deep drawing, they also showed that galvanized steel exhibited less thinning than low-carbon steel under comparable conditions. Gupta and Ravi Kumar [92] likewise identified punch speed as one of the factors influencing stamping success, although their study focused primarily on coating thickness and interface structure.

Overall, existing studies suggest that a moderate increase in forming speed is generally beneficial for improving lubrication conditions and reducing friction and coating adhesion, thereby lowering the risk of zinc powdering and coating transfer. However, excessively high speed may intensify local thinning and must therefore be optimized together with lubrication, coating type, and die-constraint conditions.

#### 4.3.4. Temperature

Temperature is a critical factor in stamping of galvanized steel sheets because it affects both coating microstructure and the tribological interaction between the sheet and the tool, thereby governing coating cracking, wear, adhesion, and zinc powdering. Qiu Xiaopan et al. [93] showed that, in galvanized 22MnB5, holding at 900 °C for 5 min increased the Fe content in the coating and promoted transformation of liquid Zn into solid alpha-Fe(Zn), which helped suppress liquid-metal-induced brittle cracking. They further reported that stamping below the melting point of the Gamma phase (782 °C) was beneficial for reducing coating cracks. Drillet et al. [94] likewise found that both macroscopic and microscopic cracks develop in zinc-based coatings during hot stamping, with microscopic cracks being particularly pronounced in friction-sensitive regions, indicating that temperature influences coating failure not only through phase evolution but also through changes in interfacial friction.

**Figure 13:**
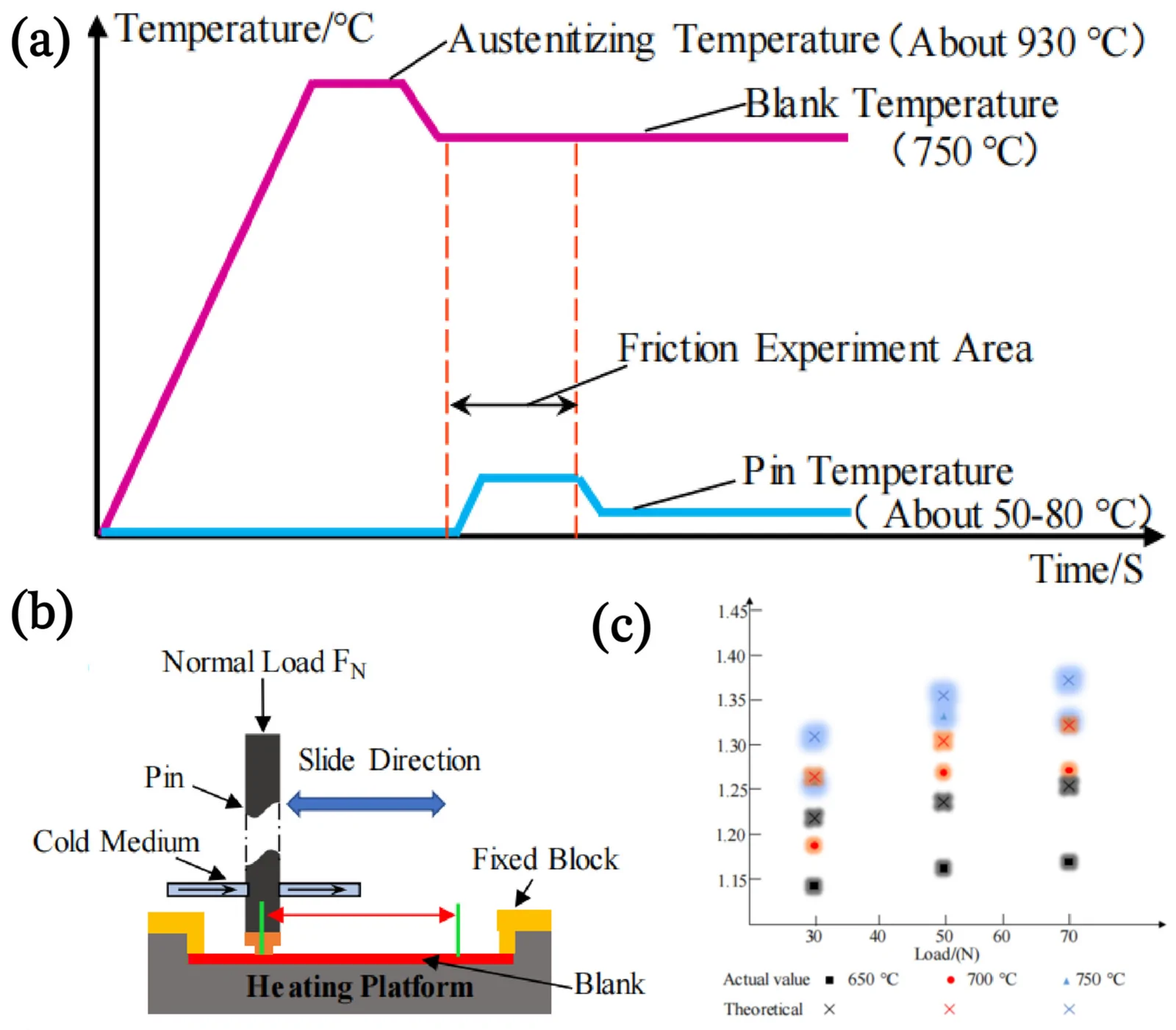
(**a**) Temperature variation curve between the plate and friction pin during the experimental process. (**b**) Structural schematic diagram. (**c**) Comparison of actual and theoretical wear volumes [86].

As shown in Figure 13, Peng et al. [86] further demonstrated, through hot-stamping tests at 650–750 °C, that temperature-dependent differences in the mechanical response of the coating and substrate significantly affect wear behavior and surface quality. Similar observations were reported by Rosiak et al. [95], who showed that coating–substrate interaction, lubrication, and hot-stamping parameters jointly determine the integrity of 22MnB5-GA coatings, while Shih [6] also noted that powdering, peeling, and sticking are particularly severe in high-contact-pressure regions such as draw beads and die corners, and are strongly affected by die temperature, lubrication, and forming conditions.

Overall, current studies indicate that temperature affects galvanized sheet stamping through two coupled mechanisms: it alters the phase constitution, brittleness, and crack susceptibility of the coating, and it modifies friction, wear, and adhesion at the sheet–tool interface. Temperature control is therefore especially important in hot-stamping applications, where thermal and tribological effects act simultaneously to promote or suppress zinc powdering depending on the processing window.

### 4.4. Applications of Finite Element Simulation

Finite element simulation has become an indispensable tool for understanding the deformation and failure behavior of galvanized coatings during sheet metal forming. Compared with experimental observation alone, numerical simulation offers distinct advantages in resolving local stress–strain evolution, interfacial load transfer, crack initiation, and tribological contact conditions that are difficult to quantify in situ. In studies on GI, GA, and related zinc-coated steel sheets, finite element methods have therefore been widely applied to analyze coating deformation and fracture, interfacial delamination, contact-pressure evolution, galling susceptibility, die wear, and multiscale friction and lubrication behavior. Existing work can be broadly classified into three categories: simulation of coating deformation, cracking, and interfacial damage; simulation of contact pressure, galling, and die wear evolution; and multiscale simulation of surface contact and friction/lubrication mechanisms.

#### 4.4.1. Simulation of Coating Deformation, Cracking, and Interfacial Damage

In the first category, finite element modeling has been extensively used to reveal deformation incompatibility and fracture mechanisms within zinc-based coatings. Parisot et al. [17] employed a crystal plasticity finite element method (CPFEM) to reconstruct a three-dimensional polycrystalline zinc coating with 34 grains in Abaqus and investigated the microstructural response of the coating under tensile loading. Their results showed that, at a macroscopic tensile strain of 2%, deformation in the zinc layer was dominated by basal slip, while the mechanical constraint imposed by the steel substrate generated a pronounced biaxial stress state within the coating. This stress localization, particularly at grain boundaries and phase interfaces, was identified as the main driving force for intergranular cracking.

Focusing on crack initiation and propagation across the multilayered coating, Ploypech et al. [18] established a two-dimensional plane–strain 2D plane strain model combined with the extended finite element method (XFEM) to simulate thermal-cooling-induced cracking and subsequent four-point bending behavior in hot-dip galvanized coatings. They found that thermal expansion mismatch among coating phases promoted crack nucleation in the brittle δ phase during cooling, whereas during bending the thicker ζ phase acted as an effective barrier to crack propagation into the outer η phase, thereby increasing the critical bending angle. In a subsequent parametric study, Ploypech et al. [19] further demonstrated that reducing the δ/(δ + ζ) thickness ratio or increasing the thickness of the outer η phase significantly improved the resistance of the coating to bending-induced cracking. These findings provide useful microstructural design guidance for improving coating ductility and crack resistance.

**Figure 14:**
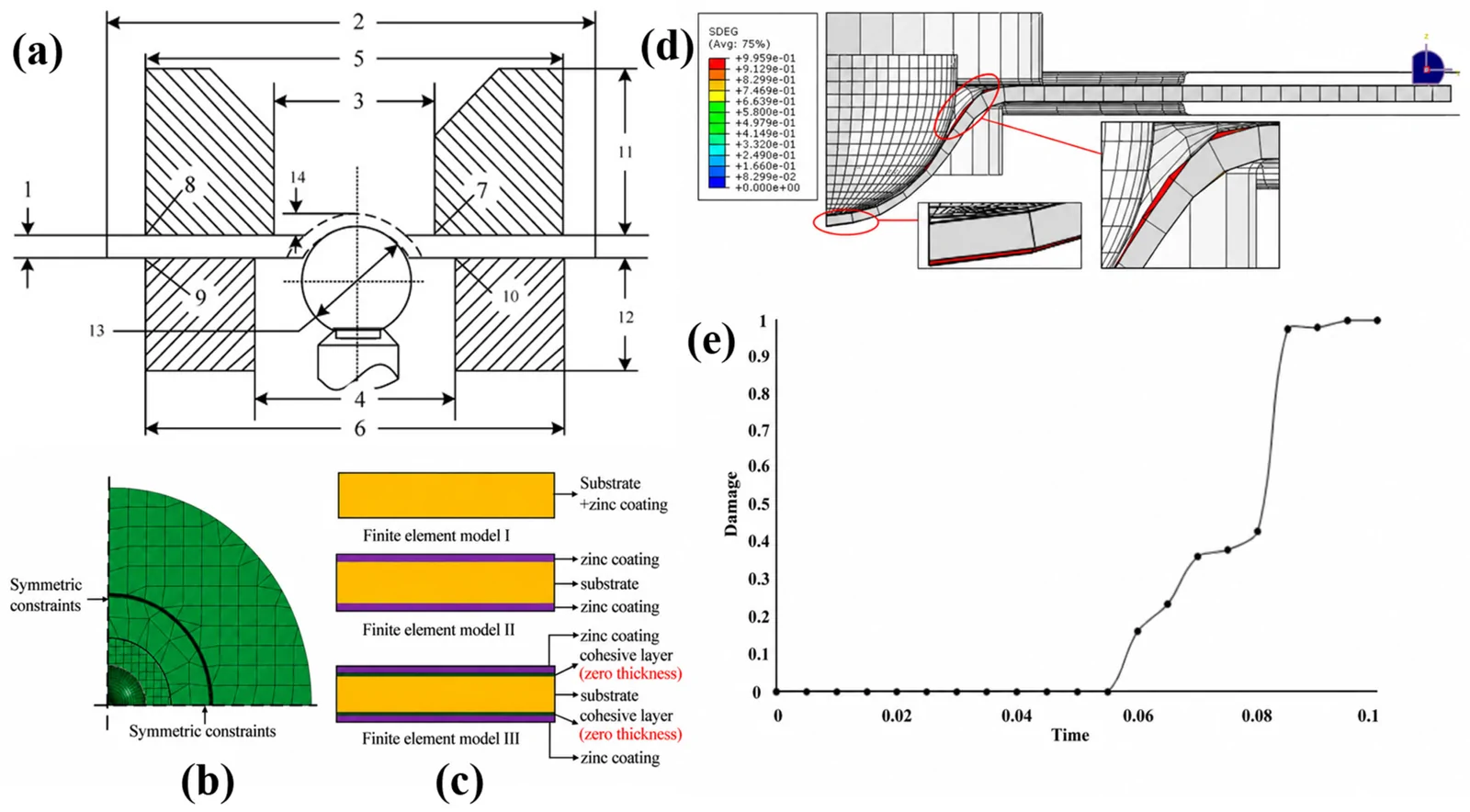
(**a**) Hemispherical part drawing model; preprocessing model of finite element analysis. (**b**) 1/4 symmetry constraints of sheet material. (**c**) Assumption of finite element model for layered structure of galvanized steel sheet; failure of cohesion element under blank holder force 50 kN. (**d**) Damage of zinc coating layers. (**e**) Damage of zinc coating layers with drawing time [81].

As shown in Figure 14a–e, Li and Long [81] developed a hemispherical punch-drawing model to investigate deformation-induced damage and interfacial delamination of hot-dip galvanized DP600 steel sheets. By exploiting the geometrical symmetry of the specimen, they established a quarter-symmetry finite element model and compared several layered representations of the sheet, including models with explicitly defined zinc-coating layers and zero-thickness cohesive elements at the coating–substrate interface. Under a blank-holder force of 50 kN, the cohesive-zone model captured the progressive evolution of interfacial damage during drawing, as illustrated by the localized damage contours in Figure 14d. The predicted damage increased gradually during the early drawing stage and then rose rapidly near the end of the stroke, eventually reaching complete failure, as shown in Figure 14e. This behavior indicates that coating delamination is a cumulative process driven by the combined effects of stretching deformation, bending over the die radius, and interfacial shear stress. The study further demonstrated that the multilayer solid-shell model incorporating cohesive elements provided improved agreement with the experimental punch-force response, highlighting the importance of explicitly considering coating–substrate interfacial failure when predicting zinc-coating cracking, powdering, and delamination during forming.

Similar approaches have also been used to compare failure modes among different zinc-based coating systems under bending and stretch-forming conditions. He et al. [20] combined nanoindentation-based constitutive parameter calibration with in situ bending strain-field simulation to investigate crack evolution in GA and electrogalvanized (EG) steel sheets. Their results indicated that the brittle Gamma phase adjacent to the steel substrate in GA coatings exhibited very high strength but negligible plastic accommodation, leading to the initiation of segmented cracks at very small bending strains. By contrast, the single-phase eta-Zn coating in EG steel deformed more compatibly with the substrate and therefore showed much better bendability. In relation to practical forming-induced coating failure, Park et al. [21] used a rigid-plastic finite element model in DEFORM-2D, calibrated by high-strain-rate tensile data, to simulate interfacial flaking behavior in GA steel during hat-bead draw forming. The simulation revealed that the region immediately downstream of the bead, especially the vertical wall, experienced the most severe combined tensile deformation and frictional shear, which led to the highest local damage accumulation and ultimately triggered extensive coating exfoliation. Likewise, GuiLi et al. [81] developed a five-layer solid-shell coupled model with zero-thickness cohesive elements in Abaqus to study cracking, powdering, and interfacial delamination in hot-dip galvanized DP600 steel during hemispherical punch stretching. Their results showed that the cohesive damage variable reached complete failure in high-strain regions such as the die radius and punch apex, successfully reproducing experimentally observed coating cracking and delamination. Among the tested modeling strategies, the five-layer cohesive model achieved the highest predictive accuracy for punch-force evolution.

Collective evidence indicates that current simulations in this category have clarified that coating damage is governed by strong mechanical incompatibility among coating phases, the steel substrate, and local forming-induced stress states. They also show that crack initiation and propagation are highly sensitive to coating architecture, phase distribution, and interface strength, which explains why different zinc-coated systems exhibit markedly different fracture behavior under otherwise similar loading paths.

#### 4.4.2. Simulation of Contact Pressure, Galling, and Die Wear Evolution

A second important application of finite element simulation concerns the evolution of contact pressure, galling, and die wear during forming. Because severe galling and tool degradation are usually concentrated in localized regions of high normal pressure and frictional shear, numerical analysis provides an effective means of identifying critical contact zones that are difficult to isolate experimentally.

**Figure 15:**
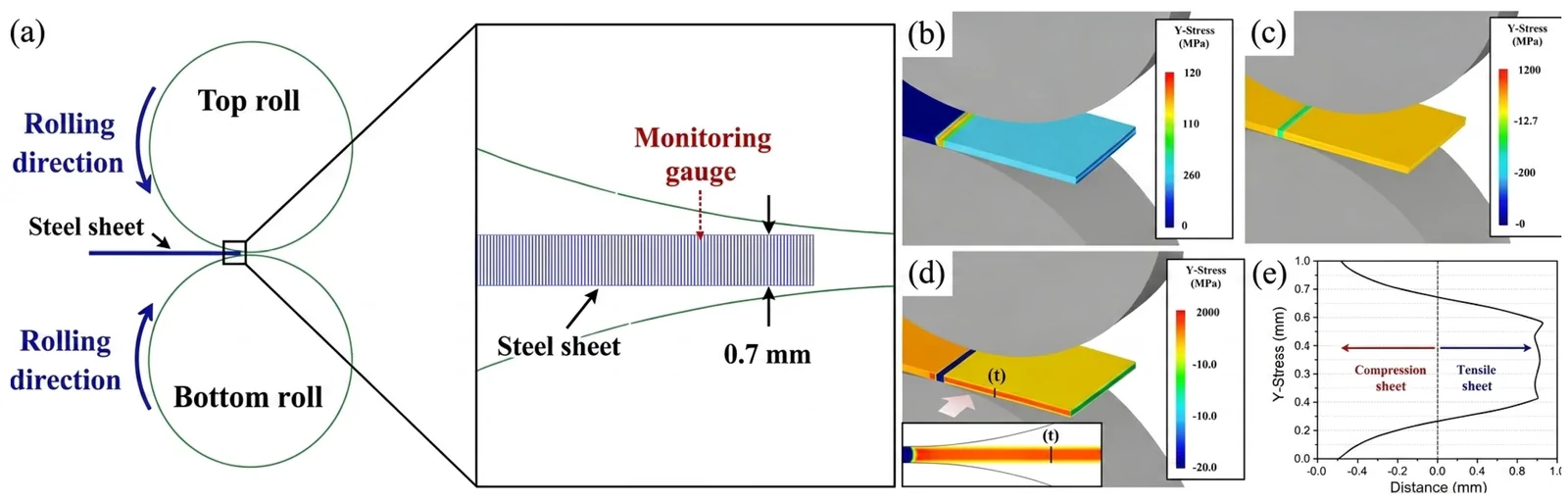
(**a**) Numerical modeling design and detailed steel sheet mesh design; simulation result of 4.0-SPM, (**b**) effective stress, (**c**) y-axis stress, (**d**) modified stress range of (**e**) (the inset indicates 2D stress distribution in the view of a pale red arrow), and (**e**) y-axis stress along the cross-section in the direction of a black arrow in (**d**) [85].

As shown in Figure 15a–e, Park et al. [85] developed a finite element model of the skin-pass rolling process to analyze the stress distribution through the thickness of galvannealed steel sheets. The model consisted of a steel sheet passing between upper and lower work rolls, with a local monitoring gauge positioned across the sheet thickness to capture the evolution of stress during rolling, as illustrated in Figure 15a. Under a rolling speed of 4.0 SPM, the simulation predicted pronounced stress gradients in the deformation zone beneath the roll–sheet contact region.

The effective-stress contour in Figure 15d indicates strong localization of deformation near the contact area, while the through-thickness distribution of the transverse stress component, shown in Figure 15e, reveals the coexistence of compressive stress near one surface and tensile stress near the opposite side. The corresponding longitudinal and transverse stress contours in Figure 15b,c further demonstrate that the coating/substrate system is subjected to a complex multiaxial stress state during rolling. Such stress heterogeneity is important for GA coatings because their brittle Fe–Zn intermetallic phases have limited capacity for plastic accommodation. Local tensile stresses can promote cracking and powdering within the coating, whereas stress concentrations and interfacial stress components may facilitate coating–substrate separation and flaking. These results highlight that coating failure during skin-pass rolling is governed not only by the nominal rolling reduction, but also by the local stress distribution generated by roll contact and through-thickness deformation incompatibility.

Pereira et al. [96] used a two-dimensional elastic–plastic plane–strain model in Abaqus/Standard to examine contact-pressure evolution along the die radius during U-bending and deep drawing. Through a numerical design-of-experiment involving 14 variables, they found that a transient contact-pressure peak occurred at the onset of forming and could exceed the steady-state pressure by more than twice. This peak was located near 70 degrees along the die radius and corresponded closely to experimentally observed regions of severe galling and wear. Moreover, the bend-radius ratio and the ultimate tensile strength of the sheet were identified as the dominant factors controlling the magnitude of this pressure peak. Hou et al. [97] further investigated the thermomechanical contact state at the die radius using a two-dimensional U-shaped forming model. Their simulations showed that the die radius was not only the region of maximum contact pressure but also the location of the highest temperature rise caused by frictional shear, making it particularly susceptible to galling initiation. Increasing blank-holder force or friction coefficient further aggravated this local contact condition and thereby accelerated interface deterioration.

These studies indicate that finite element simulation is especially valuable for locating tribologically critical regions and quantifying the local driving forces for galling and wear. In particular, they demonstrate that coating failure and tool degradation are often controlled by transient, spatially localized contact events rather than by average process conditions alone.

#### 4.4.3. Simulation of Surface Contact and Friction/Lubrication Mechanisms

Beyond macroscale contact analysis, a third research direction involves microscale and multiscale simulation of friction and lubrication behavior. Since the frictional response of galvanized sheet forming is strongly dependent on the evolution of real contact area, asperity flattening, lubricant retention, and local material transfer, multiscale approaches are particularly useful for linking surface microtopography to macroscale process response.

**Figure 16:**
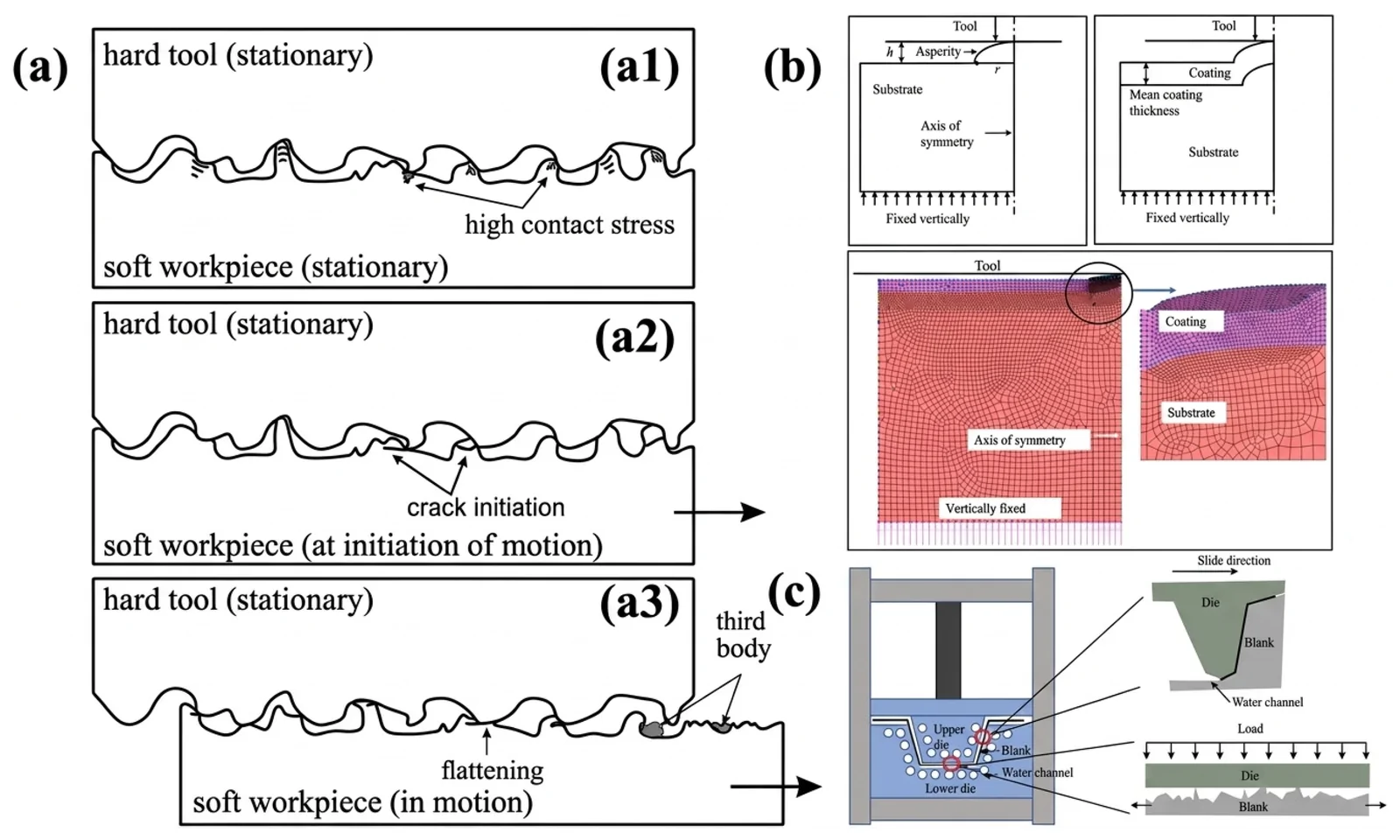
(**a**) Character of the contact between the surfaces of the hard tool and the soft workpiece: (**a1**) no movement, (**a2**) initiation of motion and (**a3**) in motion [22]. (**b**) Multiscale contact model used to predict the deformation of asperities on rough uncoated and coated surfaces under normal loading [14]. (**c**) Micro contact diagram between the die and the surface of the sheet metal [86].

As shown in Figure 16a–c, the tribological response of galvanized steel sheets is fundamentally governed by the multiscale interaction between the hard tool surface and the relatively soft-coated workpiece surface. At the asperity scale, the schematic in Figure 16a illustrates that initially stationary contact produces highly localized stress concentrations at a limited number of asperity junctions. Once sliding begins, these concentrated stresses can induce local plastic deformation, coating cracking, and asperity fracture. With continued relative motion, progressive flattening of the softer coating asperities increases the real contact area, while detached coating fragments may be entrapped at the interface and act as third-body abrasive particles [22].

To quantitatively describe this contact evolution, Shisode et al. [14] developed a multiscale semi-analytical contact model for coated sheets under normal loading, as illustrated in Figure 16b. The model explicitly considers the coating thickness, coating/substrate mechanical mismatch, asperity geometry, and substrate support. Their analysis showed that the soft zinc coating deforms readily under tool asperities, producing a larger real contact area than in uncoated steel sheets. For sufficiently large asperities or high normal loads, the deformation zone penetrates through the coating and activates the support of the underlying steel substrate, thereby changing the local contact stiffness and flattening behavior.

In addition, the microcontact model demonstrates that the actual die–sheet interface is composed of discrete contact regions rather than a uniformly loaded surface [86]. During forming, local pressure peaks at these microcontact points promote coating deformation, ploughing, wear-particle generation, and material transfer. Therefore, the real contact area and its evolution with pressure, surface roughness, coating thickness, and substrate strength are critical for predicting friction, coating damage, and tool wear. These multiscale contact mechanisms provide an essential physical basis for developing more realistic friction and wear models for GI and GA sheet forming.

Hol et al. [98] developed a multiscale friction model incorporating dynamic flattening of surface microtextures based on the Westeneng ideal-plastic flattening model and the Challen–Oxley asperity shearing theory. By performing numerical experiments on asperity deformation and integrating the resulting surface evolution into a macroscopic constitutive framework, they demonstrated that local friction coefficients could evolve dynamically between 0.13 and 0.19 depending on contact pressure and substrate strain. Building on this concept, Hol et al. [99] further incorporated boundary-lubrication effects to establish a multiscale constitutive friction model accounting for asperity flattening, junction growth, substrate straining, and multiasperity interaction. Validation in hat-section draw-bending and cross-die forming confirmed that this model could reproduce punch force-displacement responses under different blank-holder forces without repeated recalibration of a constant friction coefficient.

Under mixed lubrication conditions, Shisode et al. [100] coupled the average Reynolds equation with a global forming model to investigate the combined effects of pressure, roughness, and lubricant-film thickness on fluid–solid interaction. Their results showed that, by more realistically accounting for the flow-retarding effect of rough surfaces, the model predicted higher hydrodynamic pressure, lower solid-contact fraction, and a corresponding reduction in punch force compared with the standard Reynolds formulation. In a related study, Shisode et al. [14] developed a semi-analytical contact model supported by a finite element database of asperity indentation cases to examine the effect of coating hardness and substrate strain hardening on the real contact area of GI-coated sheets. They showed that, because the zinc coating is softer than the steel substrate, galvanized sheets develop a larger real contact area than uncoated steel under the same normal loading, while for larger asperities the deformation zone can penetrate through the coating and activate additional support from the hardened substrate.

To quantitatively evaluate the predictive capability and remaining limitations of these numerical strategies, several studies have compared FE predictions directly with experimental force–stroke responses and coating-damage observations. For example, Sener et al. [63] showed that FE simulations using simplified friction descriptions could reproduce the overall punch force–stroke trend in cupping tests of galvanized steel, although differences remained between the predicted and measured peak loads. Shisode et al. [80] further demonstrated that the agreement between simulation and experiment was sensitive to the adopted friction formulation, especially under low blank-holder-force conditions where flange wrinkling and transient contact-state changes occurred. Their results showed that incorporating boundary-friction effects improved the prediction of the forming response. In addition, Li and Long [81] compared different coating representations in hemispherical stretching of galvanized DP600 steel and found that the five-layer cohesive-zone model provided the best agreement with the experimental punch-force evolution and reproduced the experimentally observed coating cracking and interfacial delamination. These comparisons demonstrate that FE models can capture the major forming-force trends and damage locations, whereas their quantitative accuracy is strongly dependent on the treatment of coating architecture, interfacial damage, friction, and lubrication. Simplified models that neglect these coupled mechanisms may reproduce the global response but remain limited in predicting localized coating failure and its progressive evolution.

Taken together, these multiscale studies demonstrate that friction in galvanized sheet forming cannot be adequately represented by a constant coefficient alone. Instead, it should be understood as an evolving response governed by asperity deformation, coating hardness, substrate support, and lubrication state. This insight is particularly important for GI and GA systems, whose different coating architectures are expected to produce different contact-area evolution, debris formation behavior, and lubrication sensitivity during forming.

Overall, current finite element studies have substantially advanced mechanistic understanding of galvanized sheet forming across multiple length scales. At the coating scale, simulations have clarified how phase constitution, thermal mismatch, and substrate constraint control crack initiation, propagation, and interfacial delamination. At the process scale, they have identified critical regions of elevated contact pressure and frictional heating that govern galling and die wear. At the surface-contact scale, multiscale approaches have shown that friction cannot be adequately described by a constant coefficient, but should instead be interpreted as an evolving response determined by asperity deformation, coating hardness, and lubrication state. Taken together, these studies confirm that finite element simulation is not only a powerful complement to experiments, but also an essential framework for linking coating microstructure, interface mechanics, and forming performance.

Nevertheless, several limitations remain in the current literature. First, many models are established under simplified two-dimensional or idealized geometric conditions, which may not fully capture the complex three-dimensional contact states encountered in industrial forming operations. Second, although coating cracking and delamination have been simulated in considerable detail, the coupling among fracture, debris generation, transfer-layer evolution, and galling progression is still insufficiently represented. Third, most existing friction and lubrication models have been developed for generic coated or uncoated steels, whereas finite element studies specifically addressing the distinct tribological behavior of GI and GA coatings under realistic die conditions remain limited. In particular, the roles of die hardness, surface roughness, die coatings, and repeated forming cycles are rarely incorporated into integrated predictive frameworks. Future work should therefore focus on developing multiscale, multiphysics, and coating-specific simulation strategies that couple coating damage, interfacial wear, lubrication evolution, and tool-surface condition, so as to provide more reliable guidance for optimization of galvanized sheet forming in industrial applications.

## 5. Conclusions and Outlook

### 5.1. Conclusions

This review has critically examined the current understanding of wear and coating-failure behavior of galvanized (GI) and galvannealed (GA) steel sheets during sheet-metal forming by integrating findings on coating characteristics, tribological evaluation methods, damage mechanisms, and the key factors governing coating degradation. The main conclusions are summarized as follows:

(1) The tribological performance of GI and GA steel sheets is primarily governed by coating microstructure. The ductile Zn-rich outer layer of GI coatings favors plastic deformation but increases susceptibility to adhesion, smearing, and galling, whereas the harder Fe–Zn intermetallic structure of GA coatings provides greater wear resistance but promotes brittle failure, including cracking, flaking, powdering, and debris-induced abrasion.
(2) Wear and coating failure are controlled by the coupled interaction of coating properties, tool characteristics, lubrication conditions, and forming parameters rather than by any single factor. Adhesive wear, abrasive wear, coating fracture, and material transfer evolve simultaneously during forming, making coating degradation a dynamic tribological process.
(3) Existing laboratory friction tests and process-simulation methods provide complementary information for investigating coating performance. While simplified tribological tests are effective for fundamental friction and wear analysis, forming-simulation tests better represent industrial deformation conditions. Combining these approaches provides a more comprehensive understanding of coating-failure behavior.
(4) Tool-surface engineering, appropriate lubrication, and optimized forming conditions remain the most effective approaches for improving coating integrity and reducing friction and tool wear. However, their effectiveness depends strongly on the coating type and on the interactions among surface roughness, hardness, lubricant characteristics, contact pressure, and deformation history.
(5) Numerical simulation has become an important tool for understanding coating damage and guiding process optimization. Nevertheless, current models still have limited capability to capture the coupled evolution of coating fracture, debris formation, lubrication breakdown, and tool wear under realistic industrial forming conditions.
(6) The load-bearing capacity of GI and GA sheets during forming should be evaluated by the local mechanical integrity of the coating–substrate system rather than coating hardness alone. GI coatings, with their ductile Zn-rich structure, better accommodate local plastic deformation but are prone to ploughing, smearing, adhesive transfer, and galling under severe contact. GA coatings show greater resistance to mild indentation, but their brittle Fe–Zn intermetallic phases reduce deformation tolerance and increase susceptibility to cracking, fragmentation, powdering, and interfacial exfoliation under localized deformation and frictional shear. Direct comparative evidence from industrial blanking, trimming, and piercing, however, remains limited.

Based on the available evidence, improving the formability and durability of galvanized steel sheets requires an integrated tribological approach that considers coating design, interface behavior, tooling, lubrication, and process parameters simultaneously. Such a framework will provide a stronger scientific basis for optimizing industrial sheet metal forming while preserving coating integrity and extending tool life.

### 5.2. Outlook

Although considerable progress has been made, several important gaps remain in the current research on wear and coating failure of GI and GA steel sheets during sheet metal forming.

(1) Many existing studies treat tool hardness, tool roughness, lubrication, and contact pressure as independent variables, whereas these parameters interact strongly during practical forming operations. Future research should employ statistically designed experiments, such as factorial or response surface methodologies, to quantify their coupled effects. Combining laboratory friction tests with surface characterization and wear mapping under identical conditions would help identify the transition thresholds between galling, powdering, coating cracking, and delamination, thereby providing quantitative guidelines for process optimization.
(2) Comparative studies between GI and GA sheets under equivalent forming conditions remain limited. Future investigations should establish standardized testing protocols in which coating thickness, substrate grade, tool geometry, lubrication, and deformation history are carefully controlled. Such systematic comparisons would enable the development of coating-specific friction and failure criteria, predictive wear maps, and design guidelines tailored to different coated steel systems instead of relying on generalized conclusions.
(3) The influence of surface engineering on coating durability requires further clarification. Future studies should perform systematic comparisons of advanced tool-surface treatments, including PVD, CVD, nitriding, diffusion treatments, and low-adhesion coatings, while independently controlling coating thickness, surface roughness, substrate hardness, and finishing processes. Long-term cyclic forming experiments under industrial production conditions should also be conducted to evaluate coating durability, coating degradation, and tool life.
(4) Lubrication research should extend beyond conventional comparisons of lubricant types and viscosities. Future work should investigate environmentally friendly lubricants, dry-film lubricants, hybrid lubrication systems, and lubricant additives specifically designed for GI and GA coatings. Advanced experimental techniques, such as in situ observation, surface-chemical analysis, and three-dimensional surface profilometry, could be used to monitor lubricant film evolution, zinc-debris generation, debris entrainment, and third-body abrasion under realistic forming conditions, providing deeper insight into lubrication mechanisms.
(5) Greater attention should be devoted to coating damage under complex industrial forming conditions. Future studies should combine laboratory-scale experiments with pilot-scale or industrial stamping trials to investigate localized damage in critical regions such as draw beads, die radii, corners, and wall zones. Digital image correlation (DIC), three-dimensional surface scanning, and high-resolution microscopy could be integrated to characterize local deformation and coating degradation, thereby improving the reliability of laboratory evaluation methods.
(6) Future numerical simulations should evolve toward integrated multiscale and multiphysics modeling frameworks that couple coating microstructure, fracture behavior, debris generation, contact evolution, lubrication, and tool wear. These models should be validated through in situ experimental observations and detailed microstructural characterization. Furthermore, machine learning and data-driven approaches could be incorporated to optimize process parameters, predict coating failure, and accelerate the development of intelligent forming technologies for coated steel sheets.
(7) The mechanical integrity of GI and GA coatings during trimming, blanking, piercing, scratching, and edge handling remains insufficiently quantified. Future studies should distinguish between the global load-bearing capacity of the steel substrate and the local load-bearing capacity of the coating–substrate system. Standardized testing strategies combining scratch tests, cyclic sliding tests, bending tests, and controlled shearing or trimming experiments are needed. Such studies should establish coating-specific damage criteria based on critical contact pressure, critical scratch load, critical bending strain, coating-loss width at cut edges, crack density, exposed-substrate area, and zinc-debris generation.

In summary, future research should aim to establish an integrated tribological framework for GI and GA sheet forming by combining standardized experimental methods, advanced surface characterization, realistic industrial validation, and predictive numerical simulation. Such multidisciplinary approaches will provide a more comprehensive understanding of coating-damage evolution, support the design of durable tooling and lubrication systems, and ultimately improve coating integrity, reduce tool wear, and enhance the robustness and sustainability of high-performance sheet metal forming processes.

## Figures and Tables

**Table 1:** Comparison of intrinsic characteristics and tribological implications of GI and GA coatings.

Feature	GI	GA	Tribological Implication
Main coating constitution	Zn-rich outer layer	Zn–Fe intermetallic phases	Governs ductility and fracture behavior
Surface morphology	Relatively smoother	Rougher and more porous	Affects contact state and lubricant retention
Hardness	Lower	Higher	GI deforms more easily; GA resists scratching but cracks more readily
Ductility	Higher	Lower	GI better accommodates deformation
Dominant damage tendency	Smearing, adhesion, galling	Cracking, flaking, powdering	Different failure pathways

**Table 2:** Quantitative comparison of GI and GA tribological behavior.

Material	Experimental Method	Quantitative Indicators	GI Result	GA Result	Key Conclusions	Reference
TS340-GI, TS980-GI, TS340-GA, TS980-GA	Unilateral sliding friction test	COF, Ra, Rz	Ra = 0.85–0.92 μm; Rz = 5.32–6.90 μm; COF: 0.059–0.180	Ra = 1.16–1.18 μm; Rz = 8.34–8.65 μm; COF: 0.125–0.177	GA has higher COF and roughness	[10]
DP500-GI, DP600-GI, DDS-GA, AKDQ-GA	Twisted Compression Test (TCT)	COF Evolution, galloping/powdering Levels	50 MPa with no obvious galling; significant adhesive damage occurred at 170 MPa	Powder formation and coating damage occur under high pressure	GI is prone to adhesion; GA easy powdering	[24]
DC56D + Z (GI), DC56D + ZF (GA), same substrate	U-channel forming	Ry variation, coating hardness, surface damage	Coating hardness 62 HV; Ry under HRC45/52 tool is about 3.5–6.0 μm; mainly scratches	Coating hardness 296 HV; Ry ≈ 13 μm after 400 cycles at HRC35; peeling and severe scratches occur	GA has higher hardness but stronger brittleness; GI has better damage tolerance	[32]

**Table 3:** Summary of representative studies on the effect of die hardness on the surface damage of GI and GA steel sheets.

Test	Material Description		Sheet Roughness After 400 Cycles/µm			Nature of Failure	Reference
	Tool Material	Sheet Material	Die Hardness/HRC				
			35	45	52		
U-channel forming test	Mo–Cr	DX56D + Z	10.5	6	5	Galling	[31]
		DX56D + ZF	12	7	8.5	Galling	
U-channel forming test	Mo–Cr cast iron	DC56D + Z	10	6	5.5	Scratch	[48]
		DC56D + ZF	13	7	8	Powdering and exfoliation	
U-channel forming test	Mo–Cr cast iron	HDGI	9.5	6	5	Scratch	[15]
		HDGA	12	7	8	Scratches and cracks	
U-channel stamping test	Mo–Cr cast iron	GI	10	6	5	Scratch	[45]
		GA	12	6.5	7.5	Crack and scratch	
U-channel forming test	Mo–Cr cast iron	DC56D + Z	10.5	6	5	scratch	[16]
		DC56D + ZF	12.5	7	8	Exfoliation and severe scratches	

## Data Availability

No new data were created or analyzed in this study. Data sharing is not applicable to this article.
